# Differential intracellular trafficking of extracellular vesicles in microglia and astrocytes

**DOI:** 10.1007/s00018-023-04841-5

**Published:** 2023-06-30

**Authors:** Marina Pantazopoulou, Agaristi Lamprokostopoulou, Dimitra Sotiria Karampela, Anastasia Alexaki, Anastasios Delis, Audrey Coens, Martina Samiotaki, Anastasios G. Kriebardis, Ronald Melki, Stamatis N. Pagakis, Leonidas Stefanis, Kostas Vekrellis

**Affiliations:** 1 Biomedical Research Foundation Academy of Athens-BRFAA, Clinical–Experimental Surgery & Translational Research, 4, Soranou Tou Efesiou Street, 11527 Athens, Greece; 2 Biomedical Research Foundation Academy of Athens-BRFAA, Centre of Basic Research, Athens, Greece; 3 Institut Francois Jacob (MIRCen), CEA and Laboratory of Neurodegenerative Diseases, CNRS, Fontenay-Aux-Roses Cedex, France; 4 Institute for Bioinnovation, Biomedical Sciences Research Center ‘Alexander Fleming’, Fleming 34, 16672 Vari, Greece; 5 Laboratory of Reliability and Quality Control in Laboratory Hematology (HemQcR), Department of Biomedical Sciences, School of Health & Welfare Sciences, University of West Attica (UniWA), Egaleo, Greece

**Keywords:** Small extracellular vesicles (sEVs), Microglia, Astrocytes, Endocytosis, Endolysosomal pathway, Lysosome, Alpha-synuclein

## Abstract

Extracellular vesicles (EVs) have emerged as key players in cell-to-cell communication in both physiological and pathological processes in the Central Nervous System. Thus far, the intracellular pathways involved in uptake and trafficking of EVs within different cell types of the brain are poorly understood. In our study, the endocytic processes and subcellular sorting of EVs were investigated in primary glial cells, particularly linked with the EV-associated α-synuclein (α-syn) transmission. Mouse microglia and astrocytic primary cultures were incubated with DiI-stained mouse brain-derived EVs. The internalization and trafficking pathways were analyzed in cells treated with pharmacological reagents that block the major endocytic pathways. Brain-derived EVs were internalized by both glial cell types; however, uptake was more efficient in microglia than in astrocytes. Colocalization of EVs with early and late endocytic markers (Rab5, Lamp1) indicated that EVs are sorted to endo-lysosomes for subsequent processing. Blocking actin-dependent phagocytosis and/or macropinocytosis with Cytochalasin D or EIPA inhibited EV entry into glial cells, whereas treatment with inhibitors that strip cholesterol off the plasma membrane, induced uptake, however differentially altered endosomal sorting. EV-associated fibrillar α-Syn was efficiently internalized and detected in Rab5- and Lamp1-positive compartments within microglia. Our study strongly suggests that EVs enter glial cells through phagocytosis and/or macropinocytosis and are sorted to endo-lysosomes for subsequent processing. Further, brain-derived EVs serve as scavengers and mediate cell-to-glia transfer of pathological α-Syn which is also targeted to the endolysosomal pathway, suggesting a beneficial role in microglia-mediated clearance of toxic protein aggregates, present in numerous neurodegenerative diseases.

**Supplementary Information:**

The online version contains supplementary material available at 10.1007/s00018-023-04841-5.

## Introduction

Rapid and effective communication between different cell types in the central nervous system (CNS) is required for numerous functions of the brain, i.e., brain development, neural circuit maturation and homeostasis maintenance. Glial cells, including microglia and astrocytes, provide many protective roles for neurons, from immune surveillance, degradation, and antigen transfer to synaptic function and plasticity, as well as neuroprotection [1]. Neuroglia communicate with neurons and orchestrate CNS homeostasis through numerous pathways. Extracellular vesicles (EVs), containing proteins, lipids, and nucleic acids, represent an efficient way to transfer cargoes between cells in a functional manner and are categorized, according to their size and origin, to exosomes (endosomal origin), microvesicles (budding from the plasma membrane), and apoptotic bodies [2]. EV nomenclature is not clearly established yet due to the lack of consensus on specific markers of EV subtypes [3], and hence, in this article, we will refer to small EVs (< 200 nm) as sEVs. The main biological composition of sEVs is a bilayer membrane with a spheroid shape, whose structure is defined by the cell origin and is comprised of lipid elements [4], tetraspanins, integrins, etc. [5], attributing specificity in target-cell docking and subsequent internalization. This specific composition allows sEVs to cross lipids, biological membranes, as well as the blood–brain barrier (BBB). In the CNS, sEVs transfer biomolecules to neighboring cells maintaining homeostasis through the regulation of numerous cell processes, i.e., apoptosis, cell proliferation, inflammation, anti-inflammation, etc. [6].

Under pathological conditions, sEVs mediate the transfer of nucleic acids (miRNA, mRNA), lipids, and toxic proteins, contributing to the development of various diseases, i.e., neural tumors, multiple sclerosis, cerebrovascular diseases, epilepsy, traumatic brain injury, and neurodegenerative diseases [7]. Glia-derived sEVs are implicated in the pathogenesis of several neuroinflammatory and neurodegenerative diseases [6]. In neurodegenerative diseases, sEVs orchestrate the mechanisms of secretion, transmission and degradation of miRNAs and misfolded proteins, i.e., A*β*, tau, α-Synuclein (α-Syn), mHtt, etc. In Parkinson’s disease (PD), pathologic α-Syn can be secreted via sEVs [8], spreading this protein to healthy neurons, astrocytes, and microglia which in turn may induce inflammatory reactions and cell death [9, 10]. These events show that intercellular communication through sEVs plays a pivotal role in the pathogenesis of numerous brain diseases; thus, targeting such transmission pathways may represent a viable therapeutic strategy. sEVs can induce signaling in recipient cells either by direct interaction with extracellular receptors, or by fusing with the plasma membrane, or by internalization via a diversity of pathways [11]. Mechanisms of classical endocytosis include Clathrin-mediated endocytosis (CME), Caveolin-dependent endocytosis (CDE), Lipid Raft-mediated endocytosis, Macropinocytosis, or Phagocytosis. Cumulative studies have described the endocytic pathways sEVs follow, depending on their origin and target cell [12], though, in brain cells, the mechanism(s) mediating sEV uptake still remain elusive. Fitzner and colleagues demonstrated that oligodendroglia-derived sEVs are internalized by microglia through macropinocytosis [13]. In a simplified view, all extracellular materials (biomolecules, viruses, etc.) entering a cell can be sorted to early endosomes, mature to late endosomes, and can either follow the recycling path back to the plasma membrane or fuse with lysosomes [14]. The Rab5 GTPase protein is the main regulator of endosome biogenesis and trafficking [15], while Lamp1 defines late endosomes, pre-lysosomal vesicles [16], and lysosomes [17]. Although the fate of internalized sEVs is ill-defined, recent studies have shown that sEVs, akin to viruses, can highjack the endolysosomal pathway and in turn release their content within the cytoplasm [18, 19]. Altogether, the endocytic pathway(s) sEVs follow, differs among cells depending on the cell origin of sEVs, their interaction with the recipient cells as well as on the fate of sEV cargo upon internalization.

Here, we assessed the internalization and subcellular trafficking of brain-derived sEVs in mouse primary microglia and astrocytes, and hence the mechanistic pathway(s) essential for efficient cell-to-cell communication. Additionally, we sought to designate the differential processes involved in sEV uptake between the different glial cell types, that ultimately reflect their unique cellular function in the brain. Our results show that sEVs are internalized more efficiently in microglia than in astrocytes. Additionally, we provide valuable information on the role of the distinct endocytic pathway(s) and that of the endolysosomal system in the cell processing of sEVs, defining their cellular fate and destination. We examined the involvement of the actin-dependent endocytic pathways, such as macropinocytosis and phagocytosis in sEV uptake and their targeting to early and late endosomes in microglia and astrocytes. Finally, we evaluated the role of sEVs in clearing protein aggregates involved in the pathogenesis of neurodegenerative diseases using α-syn fibrils and PD as a paradigm. Interestingly, sEV-associated fibrillar α-Syn was targeted to the endolysosomal pathway for subsequent degradation in microglia. Our work unravels important intricacies with respect to the competency of the brain-resident macrophage population in pathological protein clearance and homeostasis maintenance.

## Materials and methods

### Mouse brain EV isolation

EVs were isolated from α-synuclein KO mouse brain hemispheres (CD57BL6/JOIaHsd mice, Harlan Laboratories), after removal of olfactory bulbs, cerebellum, and meninges, as previously described [20, 21]. Following dissection, brain tissues were digested with papain solution (20 Units/ml, Worthington), activated by L-cysteine (5.5 mM) in Hibernate A for 30 min, at 37 °C. Tissue was homogenized by adding two volumes of cold Hibernate A solution, and the suspension was passed through a 40-µm cell strainer and a 0.2-µm syringe filter. The filtrate was centrifuged at 300×*g* (10 min, 4 °C), and then, the supernatant was further centrifuged at 2000×*g* (10 min, 4  C), 10,000×*g* (30 min, 4 °C), and finally at 100,000×*g* (70 min, 4 °C). Following aspiration of the supernatant, the EV pellet was washed in 22–24 ml of cold phosphate-buffered saline (PBS) and centrifuged again at 100,000×*g* (70 min, 4  C). EV pellet was then diluted in 1.5 ml of sucrose solution (0.95 M), loaded on a sucrose gradient column, and centrifuged at 200,000×*g* (16 h, 4 °C). Sucrose gradient comprises of seven fractions (2–0.25 M, 1.5 ml each). Following centrifugation, all fractions were separated according to the gradient (a–g from the top to the bottom), collected individually, and diluted to PBS up to 22 ml of final volume. Following centrifugation at 100,000×*g* (70 min, 4  C), pellets were re-suspended in PBS. EV-enriched fractions were measured according to acetylcholinesterase (AchE) assays and Micro-Bradford. The content of EV sample was evaluated by the ratio of AchE in ng per μg of total protein. EVs were labeled with the lipophilic fluorophore Dil (D3911, Invitrogen). Dil was diluted in methanol (10μΜ) and sonicated for 15 min. 1 ml of EVs (100 ng/ml), diluted in PBS, with 6 µl of DiI solution (10 µM) was prepared and gently agitated at 25 °C for 1 h. Then, the mixture was centrifuged at 50,000 g, at 4 °C for 16 h. The pellet containing EVs was re-suspended in PBS and stored at − 80 °C. The amount of protein recovered in the sEV preparation is approximately 300–400 μg/brain.

### Electron microscopy

Negatively stained electron micrographs were obtained by adding 5 μl of EVs in PBS solution, onto Formvar-coated 400 mesh copper grids, fixing with 0.5% glutaraldehyde (5 μl), and staining with a 2% aqueous uranyl acetate solution (Sigma-Aldrich, USA) for 2 min. Grids with stained EVs were examined in a Philips 420 transmission electron microscope at an acceleration voltage of 60 kV and images were acquired with a Megaview G2 CCD camera (Olympus SIS, Munster, Germany).

### Western immunoblotting

Western analysis was performed as previously described with minor modifications [22]. Briefly, sEV preparations were lysed in 2% SDS, supplemented with protease inhibitors (Roche, Indianapolis, IN), probe sonicated, and incubated for 20 min at 25 °C. SDS-containing sample buffer was added, and the samples were incubated for 15 min at 42 °C. The protein lysate was resolved in 13% SDS-PAGE gel and transferred to nitrocellulose membrane. The membrane was treated with phosphate-buffered saline (PBS), containing 0.4% PFA for 30 min at 25 °C, followed by blocking with 10% skim milk/TBST for 1 h, and incubated in primary antibodies, overnight at 4 °C and in horseradish peroxidase-conjugated secondary antibodies (Invitrogen) for 2 h at 25 °C. Primary antibodies used were mouse monoclonal Flotillin-1 (Santa Cruz, sc-133153), rabbit polyclonal TSG101 (Abcam, ab125011), rabbit polyclonal VDAC (Thermo Fisher Scientific, PA1-954A), and mouse monoclonal GAPDH (Proteintech, HRP-60004).

### Proteomics

Intact EVs were lysed in SDS and the proteins were digested with Trypsin/LysC into peptides according to the Sp3 method and analyzed through LC–MS/MS using a preconcentration setup on an Ultimate3000 RSLC LC in line with a Q-Exactive Orbitrap HF-X spectrometer (Thermo Fisher Scientific) operating in DDA mode. The generated mass spectral raw files were processed using MaxQuant software (version 1.6.14.0). Trypsin was defined as protease and two missed cleavages were allowed for the database search. Minimum peptide length was set to seven amino acids. Carbamidomethylation of cysteine was defined as static modification. Acetylation of the protein N-term, oxidation of methionine, and deamidation of asparagine and glutamine residues were set as variable modifications. False Discovery Rate (FDR), for both peptides and proteins, was set to 1%. Label-free quantification (LFQ) was performed to calculate protein abundances. The “match between run” and “second peptides” options were enabled. The MS data were searched against a reviewed canonical fasta database of Mus musculus from UniProt. Venn diagrams were created using the VENNY 2.1 online tool. The mass spectrometry proteomics data have been deposited to the ProteomeXchange Consortium via the PRIDE [23] partner repository with the dataset identifier PXD038287.

### Nanoparticle tracking analysis

Mouse brain isolated EVs were subjected to nanoparticle tracking analysis (NTA) using the Nanosight NS300 instrument (NanoSight). 8 μg of EV fractions (b, c and d) were diluted with 1 ml PBS. Particle-size distribution and concentration were measured by capturing the particles undergoing Brownian movement and tracking their trajectories using laser light for particle interrogation. NTA 3.0 software was used for all analyses. Each sample was measured five times and size distribution was averaged.

### Human fibrillar α-synuclein preparation

Human wild-type α-synuclein was expressed in *E. coli* BL21 DE3 CodonPlus cells and purified as previously described [24]. Monomeric, endotoxin free (Pierce LAL Chromogenic Endotoxin Quantification Kit), α-synuclein (200 µM) in 50 mM Tris–HCl, pH 7.5, and 150mM KCl was assembled into fibrils by incubation at 37 °C under continuous shaking in an Eppendorf Thermomixer set at 600 r.p.m. for 7 days [25]. The assembly reaction was monitored by thioflavin T binding using a Cary Eclipse Fluorescence Spectrophotometer (Varian Medical Systems Inc.) set to excitation and emission wavelengths of 440 nm and 480 nm, respectively, and the nature of the fibrillar assemblies was assessed by transmission electron microscopy after negative staining with 1% uranyl acetate [25]. The resulting fibrils (PFFs, pre-formed fibrils) were centrifuged twice at 15,000 g for 10 min and re-suspended twice in PBS. Their concentration was adjusted to 350 μM in PBS. They were next fragmented to an average length of 40–50 nm by sonication for 20 min in 2 mL Eppendorf tubes using a Vial Tweeter powered by an ultrasonic processor UIS250 v (250 W, 2.4 kHz; Hielscher Ultrasonic) [26]. Fragmented fibrils were aliquoted (6 μL) in 0.5 mL Eppendorf tubes, flash frozen in liquid nitrogen, and stored at − 80 °C until use.

### Cell cultures and pharmacological treatment

Glial cells were extracted from postnatal (P1–P3) mouse brains (CD57BL6/JOIaHsd mice, Harlan Laboratories). Olfactory bulbs, cerebellum, and meninges were carefully removed. Brain hemispheres were dissected in Hank’s Balanced Salt Solution (HBSS). The brains were incubated in Dulbecco’s Modifies Eagle’s Media (DMEM) buffer with DNase (10 μg/ml final), Trypsin (T4674 Sigma, 2.5 μl/ml final) and Penicillin/Streptomycin (15,140,122; Invitrogen) for 30–45 min, at 37 °C. Trypsin was inactivated by adding DMEM with 10% Fetal Bovine Serum (10,270; Gibco). Cells were isolated by centrifugation at 300×*g* for 7 min, the supernatant was removed, and the pellet was diluted in DMEM with 10% FBS and 1% Pen/Strep. Cells were plated (~ 7,5–10 × 10^6^ cells per flask) on poly-D-lysine coated (PDL) (0.01 mg/ml) T75 flasks. Every 3 days, DMEM was renewed. After 7–9 days of dissection, flasks with mixed glial cells were agitated in orbital incubator (150 rpm, 6 h) for microglia detachment. The supernatant was harvested, and microglia cells were plated in 24-well dishes (5 × 10^4^ cells/well) with poly-D-lysine-coated glass coverslips. Astrocytic cells were harvested and plated on 24-well plates with poly-D-lysine-coated glass coverslips (3 × 10^4^ cells/well). To verify the purity of the culture, microglia were immunolabelled against Iba1. Astrocytes were stained against ALDH1L1 (pan-astrocytic marker) that detects immature, mature, and reactive astrocytes and against GFAP that preferably label reactive cells (Sup. Fig. 11). 24 h prior to treatment, cells were washed, and fresh media was added without FBS. Glia cell cultures were treated with or without sEVs (200 ng/ml) for 6 h, 24 h, and 48 h (cultures were washed 24 h post-incubation). Dynasore (ab120192, Abcam, 80μΜ), methyl-β-cyclodextrin (C4555, Sigma, 250 μM), and cytochalasin D (PHZ1063, Invitrogen, 10μΜ) inhibitors were added concomitantly with sEVs, and cells were incubated over a time course of 6 h and 24 h. EIPA (3378, Biotechne) was added at indicated concentrations 1 h prior to incubation with sEVs. For α-Syn fibrils treatments (Figs. 11, 12, Sup. Figure 10), PFFs, sEVs, and PFF + sEVs (1:2) were pre-incubated at 37 °C for 20 h. Microglia cells were single-treated with 200 ng/ml PFFs, 400 ng/ml sEVs, or double-treated with the combination (200 ng/ml PFF + 400 ng/ml sEVs) for 2 h and 6 h (cultures were washed 2 h post-addition). Transferrin, Alexa Fluor™ 488 Conjugate (ThermoFisher Scientific, T13342, 100 μM), and filipin III (Sigma, F-9765, 0.05 mg/ml) were used to assess the effect of Dynasore and Methyl-β-Cyclodextrin.

### Immunocytochemistry and confocal microscopy

Glia cells were fixed with 3.7% formaldehyde and blocking and permeabilization was performed with 0.1% Triton-X100/3% bovine serum albumin (BSA)/ 2% normal goat serum (NGS)/PBS, for 1 h at 25 °C. Cells were incubated with primary antibodies over night at 4 °C and with secondary antibodies and DAPI for 1 h at 25 °C. Primary antibodies used were rabbit monoclonal Rab5 (abcam, ab18211, 1/1000), rabbit monoclonal Lamp1 (abcam, ab24170, 1/1000), rat polyclonal Lamp1 (Santa Cruz, sc-19992, 1/300), mouse monoclonal α-Tubulin (abcam, ab7291, 1/800), rabbit monoclonal Iba1 (Wako, 019–19,741, 1/800), and mouse monoclonal D10 (Santa Cruz, sc-515879, 1/1000). Alexa Fluor™ 647 Phalloidin (ThermoFisher Scientific, A22287, 15 nM) was used to stain F-actin and MitoTracker Red CMXRos (ThermoFisher Scientific, M7512, 100 nM) to stain mitochondria.

Leica SP5 inverted confocal microscope with a HC PlanApo 63x/1.4NA oil immersion objective was used to obtain fluorescence images. This objective has theoretical resolution of 150 nm lateral and 560 nm axial at λ = 520 nm emission wavelength (green channel), and 170 nm and 640 nm, respectively, for λ = 590 nm (red channel) [27]. To improve the experimental resolution, so that smaller particle aggregates could be resolved, all images were deconvolved using the Huygens Essential software, version 21.10 (Scientific Volume Imaging, The Netherlands, http://svi.nl), using a theoretical PSF, based on image metadata and default parameters. The data sampling parameters were set following the guidelines of SVI’s PSF calculator (NyquistCalculator) at approx 60 nm in xy (pixel size) and 150 nm in Z (step size). Subsequently, both the lateral and axial resolution are expected to improve up to a factor of 2 (SVI FAQ, “How large a resolution improvement can be expected from the Huygens deconvolution?”, https://svi.nl/FaqDeconvolution).

### Imaris imaging software analysis

As described in Sup. Figure 2a, deconvolved confocal stacks were imported into the Imaris software (Bitplane), version 9.1.2, for further analysis. Segmentation of signal of interest from the respective channels (α-Tubulin, sEVs, nuclei, Rab5/Lamp1) was implemented using a. the Imaris “Surface” module, where a surface object is built around the structures of interest in a particular channel (based on the channel’s intensities that correspond to those structures of interest), and b. “masked” channels, based on a previously constructed surface. Masked channels have values zero at pixels lying outside the previously created surface (its mask) and the value of the original channel within it. To safely create the cell surface, we used the ‘’channel arithmetics’’ feature of Imaris and we merged the α-Tub channel with that of Rab5, a protein present on the plasma membrane and within the cytosol. The resulting surface is depicted as ‘’α-Tub surface’’. Besides serving as templates for building masked channels, the importance of the surface structure is that numerous statistics (e.g., volume, intensity mean, etc.) are automatically evaluated by Imaris for every particle comprising the surface. In particular, to measure total volume of sEVs per cell, puncta of sEVs per cell and the size of the individual puncta, first, a α-Tubulin surface capturing the cell structure was created, and then, a masked sEV channel retaining only the sEV channel signal within the cells was created from it. Subsequently, a new surface was created based on the masked sEV channel and the above statistics were extracted (Sup. Figure 2a i–v). To measure colocalization of sEVs with Rab5 and Lamp1, following the same procedure (α-Tubulin-masked channels to surface), respective surfaces were created. Subsequently, the signal from the three α-Tubulin-masked channels (sEVs, Rab5, and Lamp1) was masked again based on the corresponding surfaces. The formed (twice) masked channels were used for colocalization analysis by utilizing the “Coloc” module of Imaris. Manders’ coefficients were exported and colocalization was estimated according to the following equation:

$${M1}_{diff}=M1-\frac{{V}_{2}}{{V}_{ROI}},$$

where *M1* is the Manders’ coefficient 1 (percentage of above-threshold signal from channel 1, here sEVs, overlapping with above-threshold signal from channel 2, here Rab5 or Lamp1), ${V}_{ROI}$ is the total volume of the ROI (region of interest) (here the total cell volume) and ${V}_{2}$ is the volume of the above-threshold signal from channel 2 [28]. The colocalization channel and corresponding surface (yellow) were also created (Sup. Figure 2a vi–x).

To distinguish fully internalized sEVs from those lying on the plasma membrane, we used Imaris’ ‘’Distance transformation’’ function (with the “Distance outside” option) for an sEV surface created from the deconvolved sEV channel (original red channel corresponding to sEVs), computing the distance of the sEV puncta from the ‘’α-Tubulin’’ surface. Puncta with value “0” in the resulting “Minimum distance” statistic corresponds to sEVs that have entered the cell entirely (called ‘sEV inside’) and to those in the process of entering (‘sEV membrane’). These two groups are characterized by taking zero or positive values, respectively, in the “Maximum distance” statistic also created from the “Distance transformation” function. By selecting and isolating the former puncta into a new surface, the statistics of interest were extracted (Sup. Figure 2a xi–xiv).

### Statistical analysis

GraphPad Prism 9 was used for the statistical analysis. Data were assessed for normality (Shapiro–Wilk test) and two-tailed Student’s *t* test or Mann–Whitney test was used when comparing two groups, one-way ANOVA with Tukey’s correction for multiple groups, and two-way ANOVA with Tukey’s correction or multiple t-tests for multiple groups with two independent variables. Statistical significance was set as *p < 0.05, **p < 0.01, ***p < 0.001, ****p < 0.0001 and data were presented as the mean ± SEM from three independent experiments, with at least two replicates per assay. No blinding was performed and no test for outliers was conducted.

## Results

### Internalization of brain-derived sEVs by microglia and astrocytes

**Fig. 1:**
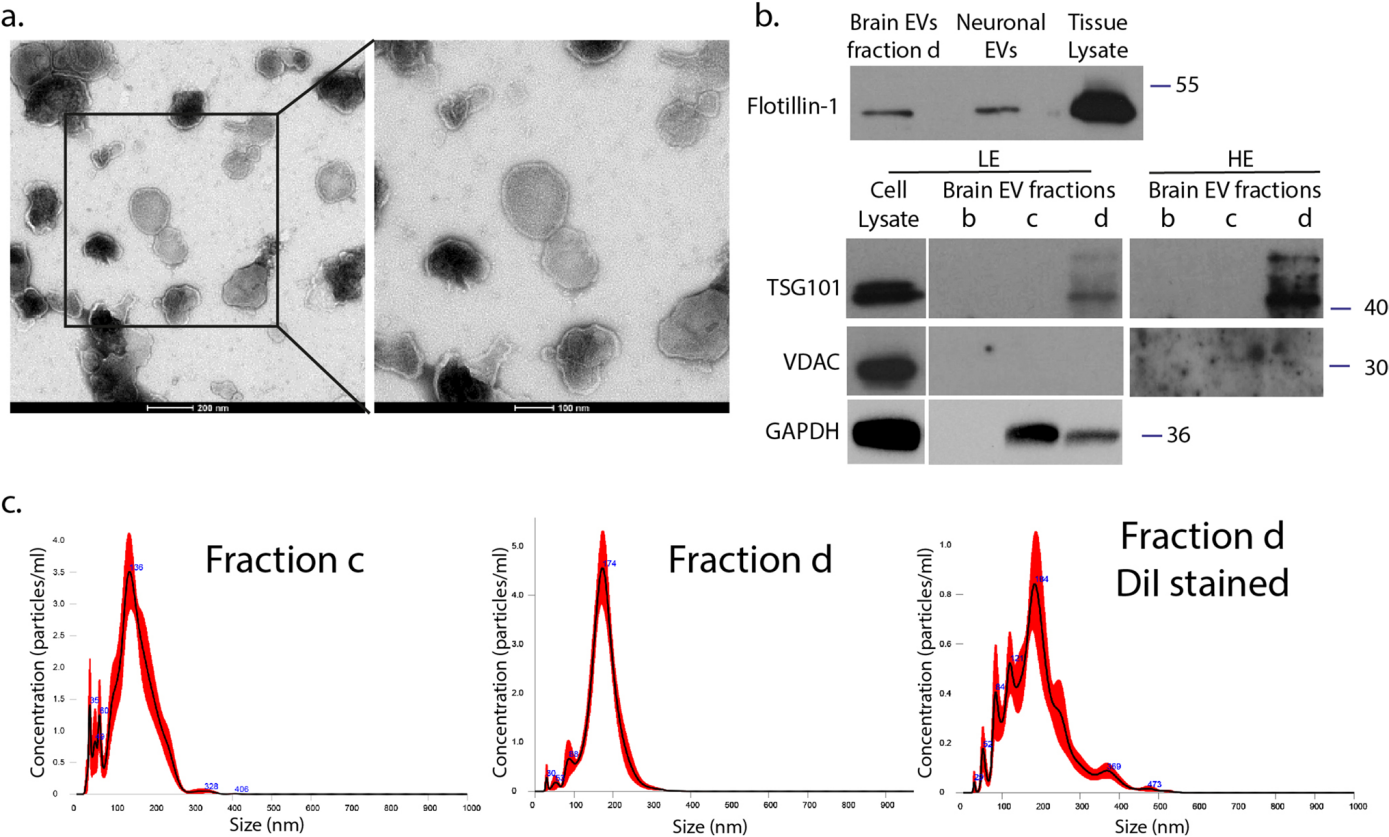
Characterization of mouse brain-derived EVs. α. Negatively stained TEM of EVs from fraction d. Scale bar 200 nm (left panel) and 100 nm (right panel). **b**. Western blotting of brain-derived EVs isolated from fraction d (lane 1), EVs derived from primary neuronal culture (lane 2), and brain tissue lysate (lane 3) (upper panel). 15 μg total protein loaded. Western blotting of different fractions (**b**, **c**, **d**) against TSG101, VDAC, and GAPDH. Cell lysate from HEK-293 cells was used as a positive control (LE: low exposure; HE: high exposure). 30 μg total protein loaded. **f**. Representative NTA distribution profile of EVs (fraction **c** and **d**, as well as DiI-stained fraction **d**), including size distribution plots and mean size of each peak

To elucidate the internalization process of EVs in mouse primary microglia and astrocytes, we isolated EVs from mouse brain using sequential centrifugations and sucrose gradient ultracentrifugation. EV enrichment in the different fractions (a-g) was quantified by measuring the activity of acetylcholinesterase. The EV-enriched fractions (fractions ‘’c’’ and ‘’d’’) were characterized using immunoblotting techniques, Electron Microscopy (EM), proteomic analysis, and nanoparticle tracking analysis (NTA). Electron Microscopy (EM) verified the size, morphology, and structure of EVs within the isolated fraction ‘’d’’ (Fig. 1a). Additionally, fraction ‘’d’’ of brain-derived EVs was shown to express flotillin-1 and TSG101, proteins generally associated with EVs, whereas non-EV markers (i.e., VDAC) were absent (Fig. 1b). Full EV protein content (fraction ‘’d’’) was identified by proteomics, resulting in a catalog of 1,120 proteins, validated with at least two different peptides. According to the “MISEV” (Minimal Information for Studies of Extracellular Vesicles) 2018 guidelines (Table 3) [3], the identified proteins were classified into five categories: (i) category 1—transmembrane or GPI-anchored proteins associated with plasma membrane and/or endosomes (blue), (ii) category 2—cytosolic proteins recovered in EVs (orange), (iii) category 3—major components of non-EV co-isolated structures (gray), (iv) category 4—transmembrane, lipid bound and soluble proteins associated with other intracellular compartments than PM/endosomes (yellow), and (v) category 5—secreted proteins recovered with EVs (green). As described before [29], we categorized 71 proteins of the 1120 proteins (Sup. Figure 1a,b). Amidst these proteins, 41.7% belong to category 1 and 42.7% to category 2. These two categories could be related to EV markers. 2.8% belong to category 3, while 12.7% to category 4. No proteins belonging to category 5 were identified. The preparation was enriched in proteins present in EVs (categories 1 and 2), containing only 2.8% of major components of non-EV co-isolated structures (category 3) and 12.7% of components of other intracellular compartments (nucleus, mitochondria, ER, and GOLGI; category 4), with the latter also present in larger EVs. Remarkably, 109 of the top 148 proteins encountered in EVs, according to Vesiclepedia, were identified in the EV-enriched fraction (Sup. Figure 1c, sheet 3). NTA analysis of EVs in fraction ‘’d’’ revealed a homogenous 173.2 ± 4.5 nm population, whereas in fraction ‘’c’’, peaks of smaller than 50 nm vesicles were detected (Fig. 1c). NTA analysis of DiI-stained EVs did not affect size distribution and measured a total particle concentration of 1.4E + 07 ± 9.95E + 06 particles/μg with a mode size of 161.8 ± 12.5 nm. Henceforth, the characterized small EVs will be referred to as sEVs.

**Fig. 2:**
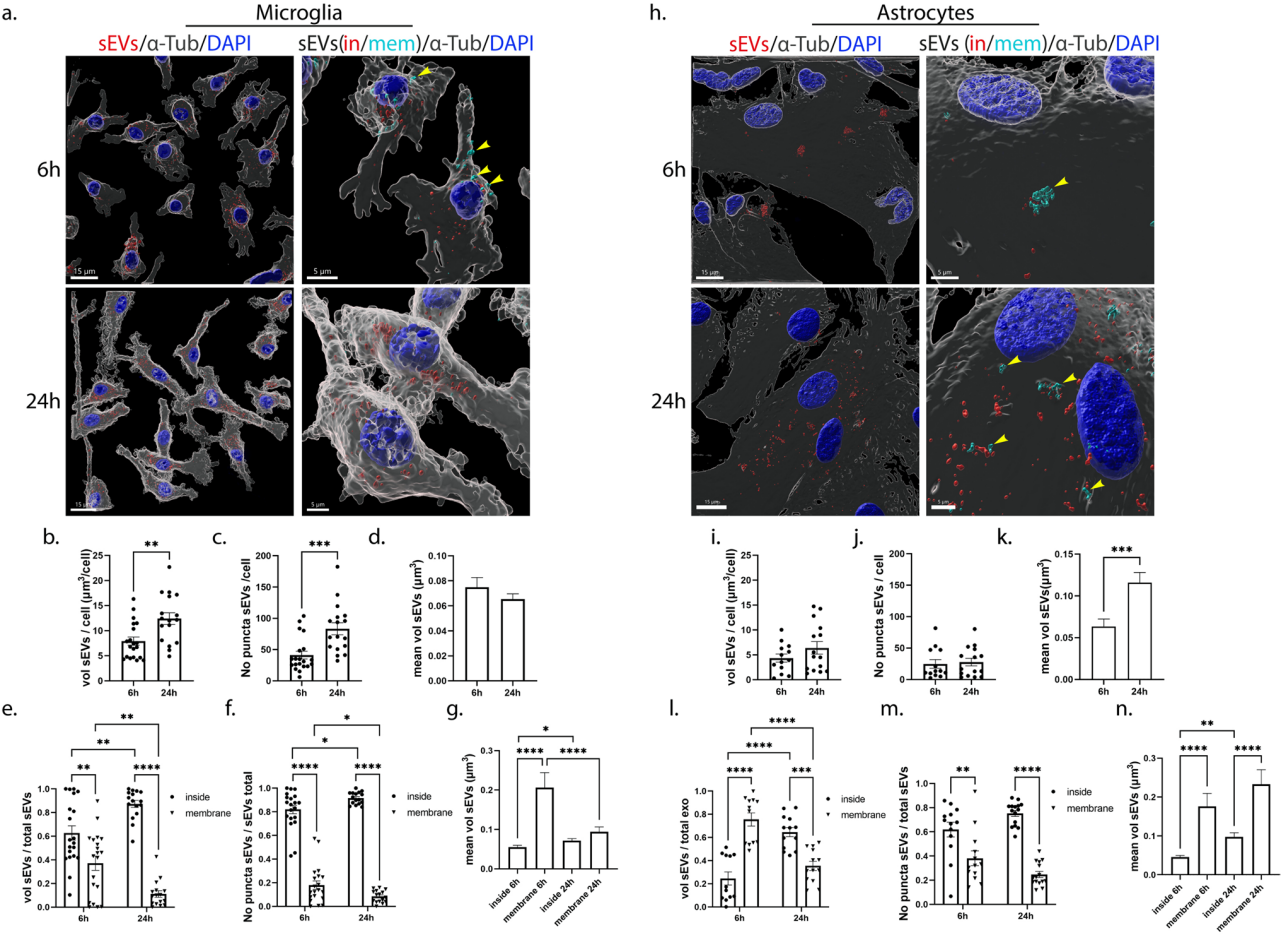
Brain-derived sEVs are internalized in primary microglia and astrocytes. Primary cells were incubated with Dil-stained brain-derived sEVs (depicted in red) for 6 h and 24 h. Cells were washed, and internalization of sEVs was monitored 6 h and 24 h post-incubation. Cells were fixed and immunostained with an antibody against α-Tubulin (α-Tub) (gray), while cell nuclei were stained with DAPI (blue). Confocal images were deconvolved and analyzed with the Imaris Imaging software. sEVs were defined as cytoplasmic (sEVs in, red) or membranous (sEVs mem, cyan blue) using the ‘’Distance transformation’’ module of the Imaris Imaging software, computing the distance of the sEV puncta from the ‘’α-Tubulin’’ surface. Representative Imaris images depict the internalization of sEVs masked with the α-Tubulin surface (left panel, scale bar 15 μm) and in/mem sEVs (right panel, scale bar 5 μm) in microglia (**a**) and astrocytes (**h**), 6 h and 24 h post-addition. ‘’sEVs mem’’ were depicted with arrowheads. Graphs show the total volume of internalized sEVs per cell (**b**, **i**), the number of puncta per cell (**c**, **j**), the mean volume of sEVs (**d**, **k**), the ratio of the volume of sEVs (in/mem) per total volume (**e**, **l**), the ratio of the number of puncta (in/mem) per total number (**f**, **m**), and the mean volume of sEVs (in/mem) (**g**, **n**), in microglia and astrocytes, respectively. Data are presented as the mean ± SEM of minimum three independent cell preparations, with at least two replicates per assay; Student's *t* test was used for (**b**), (**d**), (**i**), and (**k**), Mann–Whitney test for (**c**) and (**j**), one-way ANOVA with Tukey’s correction for (**g**) and (**n**), two-way ANOVA with Tukey’s correction for (**e**) and (**m**), and multiple *t* test for (f) and (m). Statistical significance was set as *p < 0.05, **p < 0.01, ***p < 0.001, ****p < 0.0001

To follow the internalization of sEVs in primary glial cells, sEVs were prestained with DiI, a fluorescent lipophilic cationic dye. Labeled sEVs were added to primary glia cultures (200 ng/ml, measured as sEV protein concentration per ml of medium) and washed off after 24 h of incubation, and uptake was monitored 6 h, 24 h and 48 h post-addition. Following immunocytochemistry and confocal microscopy, image analysis was performed using the Imaris Imaging software (Sup. Figure 2a). Additionally, to validate that DiI stains sEVs and not intracellular membranes, the colocalization of the labeled sEVs with a cellular marker specific for EVs (TSG101) was assessed and showed that 60% of sEVs are TSG101 positive (Sup. Figure 2b). To evaluate sEV uptake at different time points and conditions, we measured the total volume and number of puncta of sEVs per cell. Values corresponding to the amount of sEVs internalized per cell, as well as the size of the sEV puncta, revealing the size distribution and intracellular processing, were derived from those measurements. sEV puncta were defined as clusters of sEVs. Our data showed that sEVs were internalized by both astrocytes and microglia but at a different rate (Fig. 2). As shown in Fig. 2, after 6 h of incubation, microglia cells had uptaken sEVs (Fig. 2a,b) more efficiently than astrocytes (Fig. 2h,i); 7.01 μm^3^ and 4.34 μm^3^ of sEVs per cell, in microglia and astrocytes, respectively. Additionally, total puncta per cell significantly increased after 24 h of incubation in microglia (Fig. 2c) but not in astrocytes (Fig. 2j). In microglia, the size of the individual puncta remained the same (Fig. 2d), whereas, in astrocytes, it increased over time (Fig. 2k). In the absence of sEVs or when incubating cells with the DiI solution, remaining in the supernatant after centrifugation to separate stained sEVs and excess DiI, no signal was observed (data not shown). Next, and to distinguish the sEVs that have fully entered the cells from those that were in the process of entering, we used the ‘’Distance transformation’’ function of the Imaris software, to compute the distance of the sEV puncta from the ‘’α-Tubulin’’ surface (Sup. Figure 2a xi–xiv). Hence, we divided sEVs intersecting the cell (‘’sEV total’’) into two separate fractions corresponding to those localized within the cytoplasm (sEV inside, henceforth ‘‘sEV in’’) and those localized on the plasma membrane, in the process of entering (sEV membrane, henceforth ‘’sEV mem’’). We measured the proportion of sEVs localized in the cytoplasm (sEV in) or on the plasma membrane (sEV mem) by calculating the ratio of the sEV in or mem (in/mem) volume and number of puncta to total volume and puncta, respectively. Our data suggested that almost 60% of sEVs were internalized (sEV in) by microglia, with 40% remaining on the plasma membrane (Fig. 2e), whereas, in astrocytes, this process was slower with over 70% remaining on the plasma membrane (Fig. 2l) after 6 h of incubation. 24 h post-addition, most sEVs were completely internalized (sEV in) by microglia, while they also grew in size (Fig. 2e,g), whereas in astrocytes, 40% still resided on the plasma membrane (Fig. 2l). When the number of sEV puncta was measured in microglia, 80% of sEV puncta were internalized (sEV in) at 6 h (Fig. 2f). The puncta localized on the plasma membrane appeared larger than those present inside the cells 6 h post-incubation, and their size decreased over time when most of them had fully entered the cell (Fig. 2g). In astrocytes, at 6 h, 40% of the puncta were membrane-associated and much larger in size than those present inside (Fig. 2m,n). Their size remained unchanged over time, whereas the size of sEVs localized inside the cells increased (Fig. 2n), consistent with the overall increase of size of sEVs between 6 and 24 h.

When the medium was changed after 24 h of incubation, the total volume of sEVs remained the same between 24 and 48 h post-addition with most sEVs residing inside both glial cell types (Sup. Figure 3a,b,e,f,h,i,l,m). Since the DiI fluorophore is stable even in acidic environments, no alteration in the total volume was expected. In microglia, the size of the individual puncta increased 48 h post-addition, due to the increase in size of the few membranous puncta (Sup. Figure 3d,g). In astrocytes, the size of sEVs localized on the plasma membrane decreased at 48 h (Sup. Figure 3n) as it did in microglia after 24 h of incubation. Overall, both glial cell types uptake brain-derived sEVs; however, this process is faster in microglia than in astrocytes.

### Endocytic trafficking of sEVs in microglia and astrocytes

**Fig. 3:**
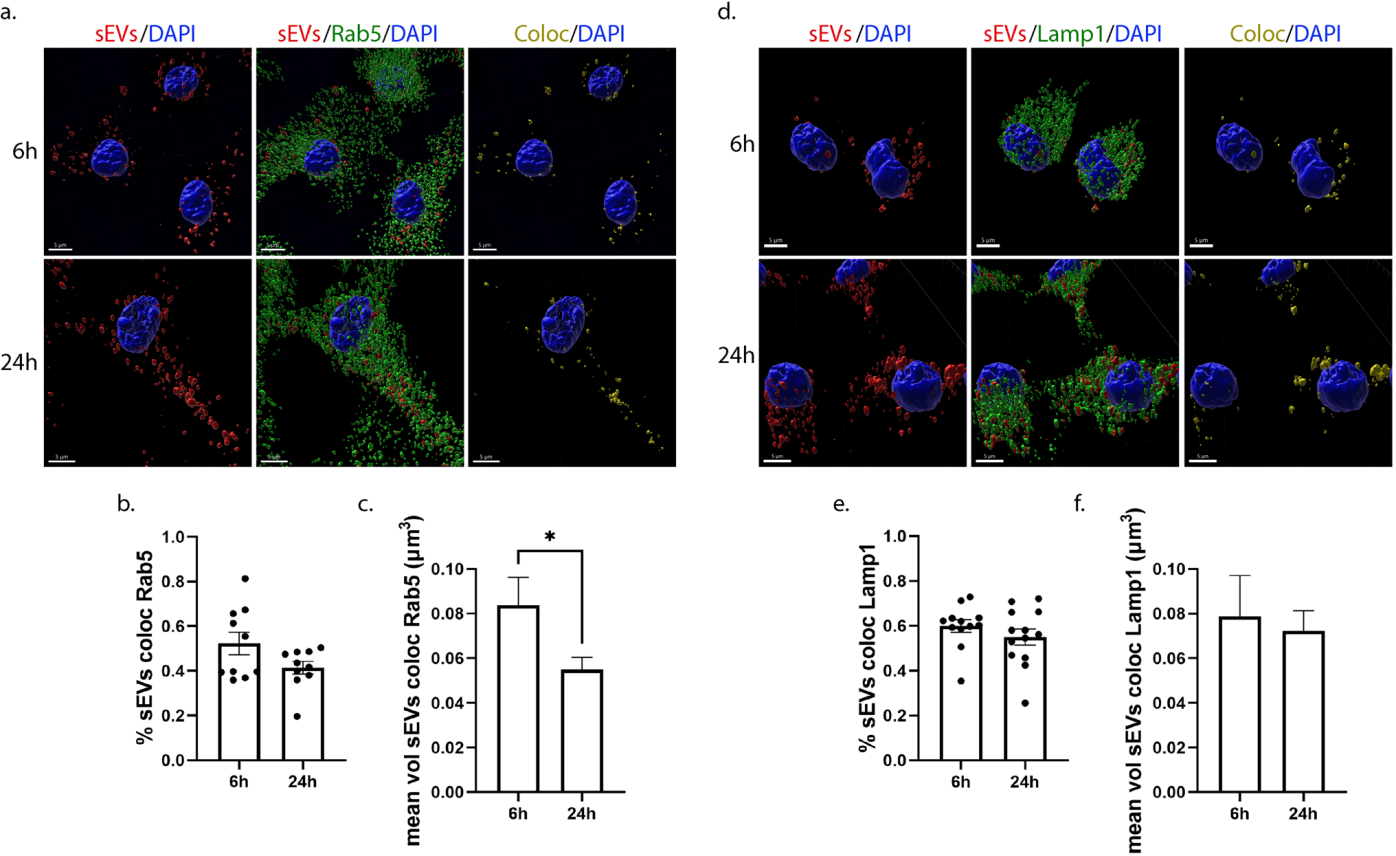
sEVs when uptaken by primary microglia follow the endocytic pathway and are colocalized with Rab5 (early endosomes-EE) and Lamp1 (late endosomes-LE/Lysosomes). Microglia cells were incubated with Dil-labeled sEVs (red), for 6 h and 24 h. Colocalization of sEVs with Rab5 and Lamp1 was monitored at 6 h and 24 h post-treatment. Cells were fixed and immunolabeled for Rab5 or Lamp1 (green), α-Tubulin (α-Tub) (gray), and DAPI (blue), detecting cell nuclei. Representative Imaris images depict colocalization between internalized sEVs and the endocytic markers, Rab5 (**a**–**c**) and Lamp1 (**d**–**f**), after 6 h and 24 h of incubation. The colocalization channel and surface (yellow) were built using the Imaris imaging software. Scale bar 5 μm. Graphs show colocalization between sEVs and Rab5/Lamp1 (Manders’ colocalization coefficient) after 6 h and 24 h (**b** and **e**) of treatment as well as the mean volume of puncta colocalized with Rab5 (**c**) and Lamp1 (**f**) at the different time points. Data are presented as the mean ± SEM of minimum three independent cell preparations, with more than 80 cells measured; Student's t test was used, and statistical significance was set as *p < 0.05, **p < 0.01, ***p < 0.001, ****p < 0.0001

Next, we wished to elucidate the endocytic pathway sEVs follow post-internalization in primary glial cells. Thus, we used two endocytic markers of the early and late endocytic pathway. Rab5, a cytosolic protein localized at the level of endosomal membranes and to a lesser extent to the plasma membrane, is a key regulator for the formation of early endosomes (EE) [15], and LAMP1 (lysosomal-associated membrane protein 1) is associated with the late endosomal, pre-lysosomal, and lysosomal pathway (LE/Lysosome) [16, 17]. Therefore, we investigated whether sEVs are sorted in EE and LE/Lysosome 6 h, 24 h, and 48 h following their addition to cells. To follow the trafficking of sEVs, Dil-labeled sEVs were added to primary glial cultures, washed off after 24 h of incubation, and the cells were fixed and immunostained with antibodies against Rab5 or Lamp1. We examined the colocalization of sEVs with the endosomal markers, by performing immunofluorescence and confocal microscopy followed by Imaris analysis that permitted 3D reconstruction analysis of pixel colocalization between Dil-stained sEVs and endosomes (EE, LE/Lysosome). Quantitative analysis indicated that in microglia, approximately 55% of internalized sEVs colocalize with Rab5 at 6 h and 40% at 24 h post-addition, with the size of colocalized puncta decreasing over time (Fig. 3a-c). 24 h after washing the cells (48 h post-treatment), the percentage of colocalization between sEVs and Rab5 remained the same as did the size of colocalized puncta (Sup. Figure 4a–c). Additionally, 60% of sEVs colocalized with Lamp1, and this percentage did not change significantly over time. The size of the colocalized puncta also remained the same throughout the experimental timeframe (Fig. 3d–f, Sup. Figure 4d–f).

**Fig. 4:**
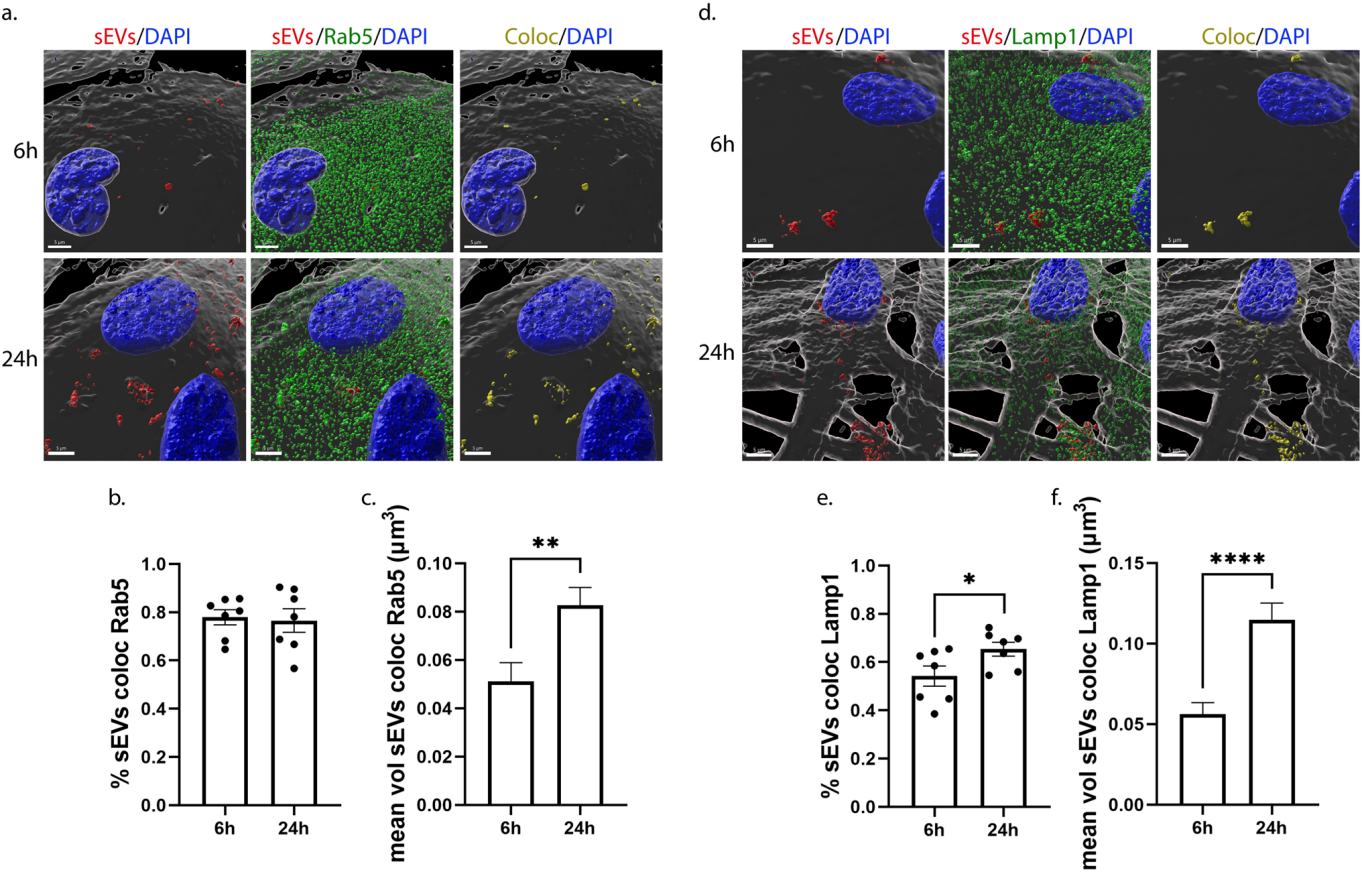
sEVs are targeted to the endocytic pathway and are colocalized with Rab5 (EE) and Lamp1 (LE/Lysosomes) following addition on primary astrocytes. Cells were incubated with Dil-stained sEVs (red) for 6 h and 24 h, and colocalization of sEVs with Rab5 and Lamp1 was measured 6 h and 24 h post-treatment. Cells were fixed and immunostained for Rab5 and Lamp1 (green), α-Tubulin (α-Tub) (gray), and DAPI (blue). Representative Imaris images depict colocalization between internalized sEVs and the endocytic markers, Rab5 (**a**–**c**) and Lamp1 (**d**–**f**), after 6 h and 24 h of treatment. The colocalization surface is depicted in yellow. Scale bar 5 μm. Graphs show the colocalization between sEVs and Rab5/Lamp1 (Manders’ colocalization coefficient) after 6 h and 24 h (**b** and **e**) of treatment as well as the mean volume of puncta colocalized with Rab5 (**c**) and Lamp1 (**f**). Data are presented as the mean ± SEM of minimum three independent cell preparations, with more than 60 cells measured; Student's t test was used, and statistical significance was set as *p < 0.05, **p < 0.01, ***p < 0.001, ****p < 0.0001

In astrocytes, approximately 75% of sEVs colocalized with Rab5 at both time points examined (Fig. 4a,b), while 55% of sEVs colocalized with Lamp1 at 6 h and this colocalization slightly but significantly increased after 24 h of treatment (Fig. 4d,e). The size of colocalized puncta increased over time in both cases as sEVs resided in early or late endosomal compartments (Fig. 4c,f). In contrast, after washing the cells (48 h post-treatment), the percentage of colocalization between sEVs and Rab5 decreased to 45%, as did the volume of colocalized puncta (Sup. Figure 5a–c). No differences were observed concerning the colocalization of sEVs with LE/lysosomes; however, the size of colocalized puncta decreased over time, probably due to fusion processes with the lysosome (Sup. Figure 5d–f). To ensure that the proportion of sEV colocalization with endosomes is accurate, a MitoTracker probe was used as a negative control. The lack of colocalization with Rab5 and Lamp1 suggested that our method was robust, since specific colocalization was not observed (Sup. Figure 5g–i). Our data indicate that sEVs are targeted to the endolysosomal pathway in both glial cell types; however, the distribution to EE or to LE/Lysosomes differs when comparing microglia and astrocytes.

### Effect of the dynamin-dependent endocytic pathway inhibition on the uptake and endocytic trafficking of sEVs in primary glia cells

**Fig. 5:**
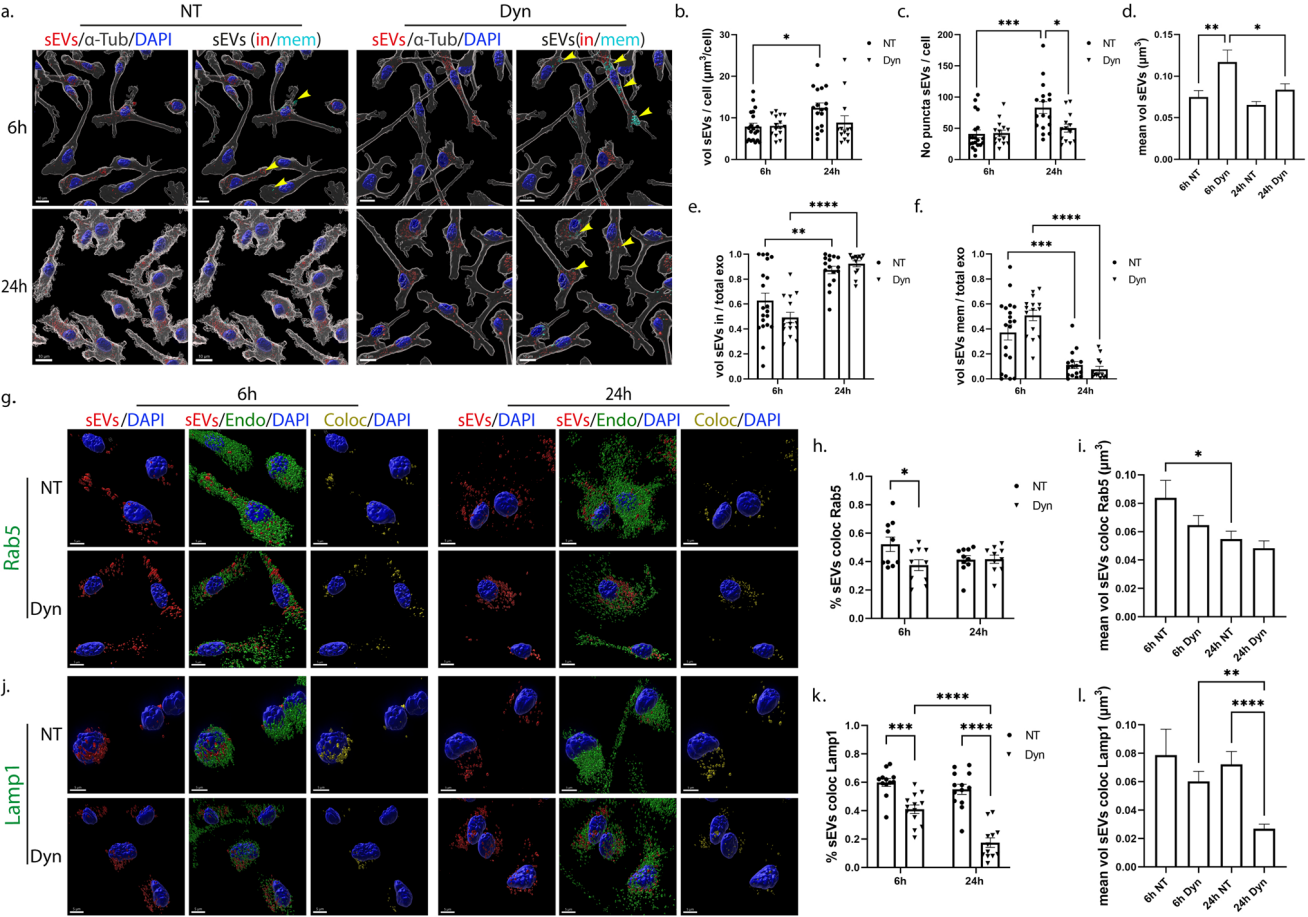
Uptake and endocytic trafficking of sEVs in primary microglia following inhibition of the dynamin-dependent endocytic pathway. Cells were incubated with Dil-labeled sEVs (red), in the absence (NT) or presence of Dynasore (Dyn) for 6 h and 24 h. Cells were fixed and immunostained against α-Tubulin (α-Tub) (gray) and DAPI (blue). Representative Imaris images depict the internalization of sEVs masked with the α-Tubulin surface and in/mem sEVs without (NT) or with Dynasore (Dyn) at 6 h and 24 h (**a**) of incubation with sEVs. Scale bar 10 μm. Graphs show the total volume of internalized sEVs per cell (**b**), the number of sEV puncta per cell (**c**), the mean volume of internalized sEVs (**d**), the percentage of sEV volume, inside (**e**) or on the membrane (**f**) per total volume of sEVs. The endocytic trafficking of sEVs was evaluated by measuring the colocalization of sEVs with Rab5 (**g**–**i**) and Lamp1 (**j**–**l**). ‘’sEVs mem’’ were depicted with arrowheads. Scale bar 5 μm. Graphs show colocalization between sEVs and Rab5 (**h**) or Lamp1 (**k**) and the mean volume of colocalized puncta (**i** and **l**, respectively) at different time points. Data are presented as the mean ± SEM of minimum three independent cell preparations, with at least two replicates per assay; one-way ANOVA with Tukey’s correction was used for (**d**), (**i**), and (**l**), two-way ANOVA with Tukey’s correction for (**b**), (**e**), (**f**), (**h**), and (**k**) and multiple t test for (**c**). Statistical significance was set as *p < 0.05, **p < 0.01, ***p < 0.001, ****p < 0.0001

Dynamin is the key regulator of clathrin- and caveolin- dependent endocytosis [30]. To assess the role of these pathways in the internalization and intracellular trafficking of sEVs, we used Dynasore (Dyn), a pharmacological reagent that inhibits dynamin [31]. DiI-labeled sEVs (200 ng/ml) were added to primary cultures in the absence (NT) or presence of Dyn and uptake was monitored at 6 h and 24 h post-addition. Immunocytochemistry and confocal microscopy, followed by Imaris analysis, showed that sEVs were taken up by microglia, and no difference was observed in the total volume of sEVs per cell between NT and Dyn-treated cells (Fig. 5a,b). Interestingly, in Dyn-treated cells, the total volume and the number of puncta remained comparable between time points (Fig. 5b,c). There was a statistical, albeit slight increase in the size of puncta upon Dyn treatment, 6 h post-incubation (Fig. 5d), but no differences were reflected in the in/mem puncta size (Sup. Figure 6c,d). Likewise, no differences were observed in the proportion of in/mem sEVs (volume and puncta) or in their size between NT and Dyn-treated cells, at any time point (Fig. 5e,f, Sup. Figure 6a–d). After 24 h of incubation, the majority of sEVs (volume and puncta) had been efficiently targeted from the plasma membrane to the cytoplasm in both conditions.

To gain further insight into the endocytic trafficking of sEVs when dynamin is inhibited, colocalization with EE and LE/Lysosomes was assessed. Our data indicate that colocalization of sEVs with Rab5 (Fig. 5g–i) and Lamp1 (Fig. 5j–l) decreased by approximately 20% following Dyn treatment at 6 h. The percentage of colocalization with Lamp1 and the size of Lamp1-colocalized puncta decreased over time. Altogether, sEVs enter microglia in a clathrin- and caveolin-independent manner, while their targeting to and processing through the endolysosomal pathway is partially hindered upon Dyn treatment.

**Fig. 6:**
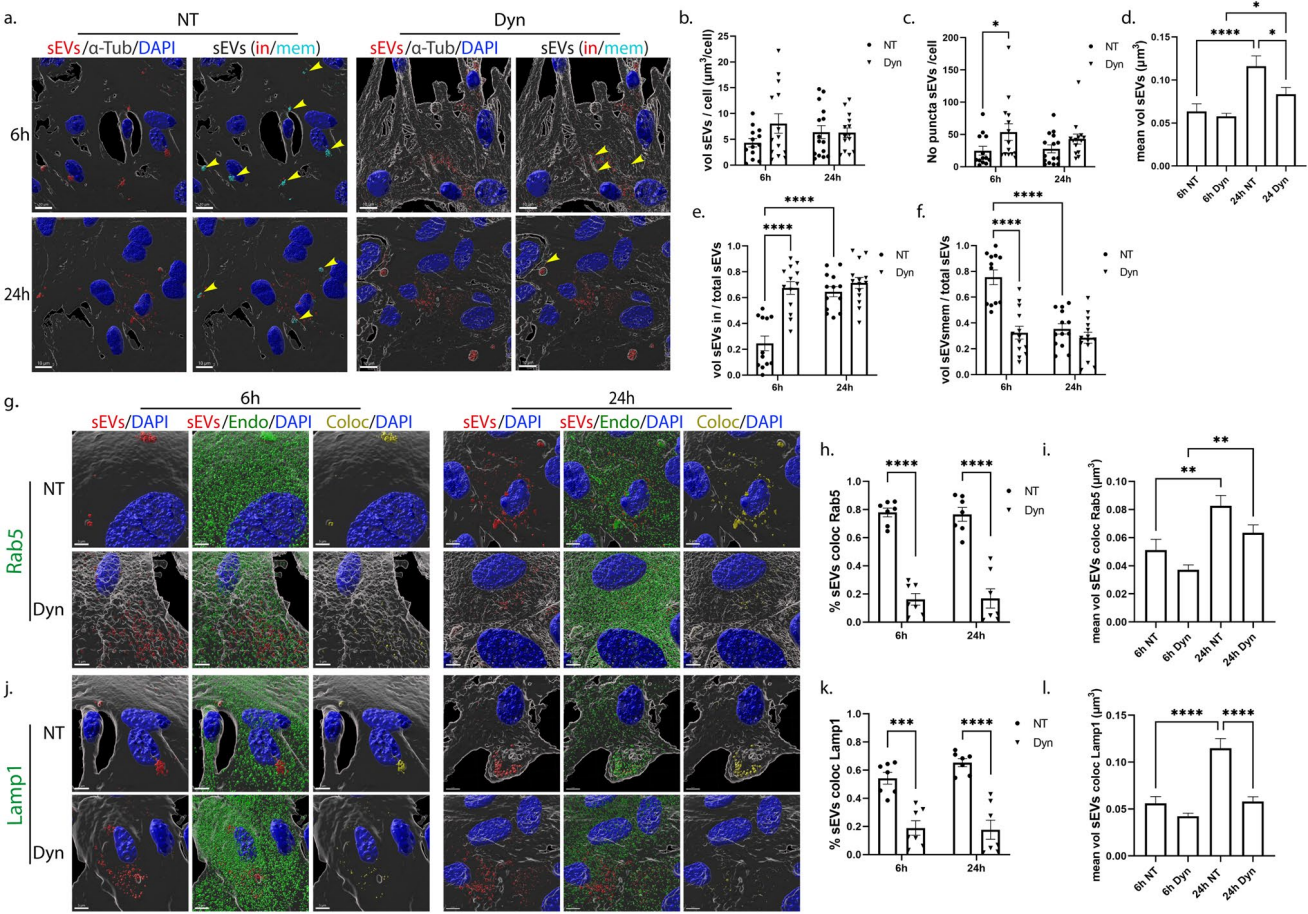
Internalization and endocytic trafficking of sEVs in primary astrocytes following Dynasore treatment. Cells, incubated with Dil-labeled sEVs (red), in the absence (NT) or presence of Dynasore (Dyn) for 6 h and 24 h, were fixed and immunostained against α-Tubulin (α-Tub) (gray) and DAPI (blue). Representative Imaris images depict the internalization of sEVs masked with the α-Tub surface and in/mem sEVs in cells treated without or with Dyn at 6 h and 24 h (**a**). ‘’sEVs mem’’ were depicted with arrowheads. Scale bar 10 μm. Graphs show the total volume of internalized sEVs per cell (**b**), the number of sEV puncta per cell (**c**), the mean volume of internalized sEVs (**d**), the percentage of sEV volume, inside (**e**) or membrane (**f**) per total volume, after 6 h and 24 h of treatment. The endocytic trafficking of sEVs was monitored by measuring the colocalization of sEVs with Rab5 (**g**–**i**) and Lamp1 (**j**–**l**). Scale bar 5 μm. Graphs show the colocalization between sEVs and Rab5 (**h**) or Lamp1 (**k**) and the mean volume of colocalized puncta (**i** and **l**, respectively) at different time points. Data are presented as the mean ± SEM of minimum three independent cell preparations, with at least two replicates per assay; one-way ANOVA with Tukey’s correction was used for (**d**), (**i**), and (**l**), two-way ANOVA with Tukey’s correction for (**b**), (**e**), (**f**), (**h**), and (**k**) and multiple t test for (**c**). Statistical significance was set as *p < 0.05, **p < .01, ***p < 0.001, ****p < .0001

In astrocytes, Imaris analysis indicated that upon Dyn treatment, the total volume of sEVs internalized was the same as in control NT conditions (Fig. 6a,b). However, increased number of internalized puncta (sEV in) was detected after 6 h of incubation (Fig. 6c). There was a statistically significant increase in the size of puncta over time in NT cells, and to a lesser extent in Dyn-treated cells (Fig. 6d). Comparing the fractions, in/mem, between the treatments, we observed that less sEVs were confined on the plasma membrane in Dyn-treated cells (ratio of volume and puncta), with more than 60% residing within the cytoplasm, 6 h post-addition (Fig. 6e,f, Sup. Figure 6f,g). The size of membrane-associated puncta is smaller at both time points and those inside remain the same in size over time compared to control NT cells (Sup. Figure 6h,i). Assessing the intracellular distribution of sEVs in the endocytic compartments, it appears that sEVs escape the endocytic pathway to a great extent as evidenced from the 60% and 40% decrease in sEV colocalization with Rab5 and Lamp1, respectively (Fig. 6g,h,j,k). Additionally, the size of Rab5-colocalized puncta increased overtime, in a treatment-independent manner (Fig. 6i), and that of Lamp1-colocalized puncta remained unaltered overtime in Dyn-treated cells (Fig. 6l). As expected, uptake of transferrin, a marker of CME, was inhibited upon Dyn treatment in both cell types (Sup. Figure 6e,j) Our data suggest that sEV internalization in Dyn-treated astrocytes was faster at early time points, with less sEVs residing on the plasma membrane, whereas no effect was depicted in microglia. In both cell types, sEVs failed to a greater or lesser extent to localize in EE and LE/Lysosomes.

### Uptake and endocytic trafficking of sEVs in primary glia cells following inhibition of lipid raft-mediated endocytosis

**Fig. 7:**
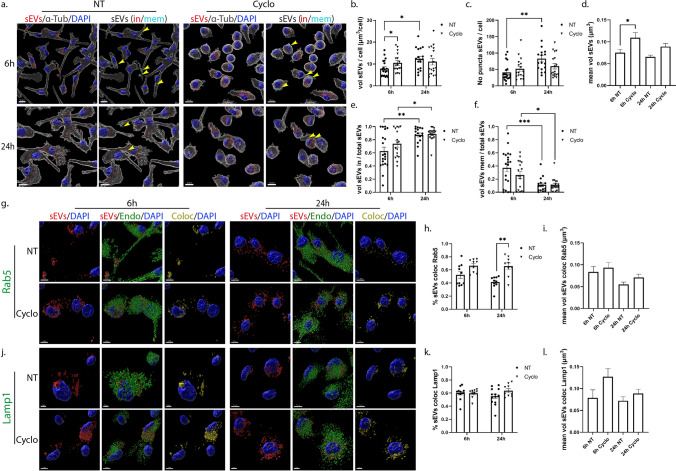
Uptake and endocytic trafficking of sEVs in primary microglia upon depletion of cholesterol with methyl-β-cyclodextrin. Cells, treated with Dil-labeled sEVs (red), in the absence (NT) or presence of methyl-β-cyclodextrin (Cyclo) for 6 h and 24 h, were fixed and immunostained against α-Tubulin (α-Tub) (gray) and DAPI (blue). Representative Imaris images depict the internalization of sEVs and in/mem sEVs without or with methyl-β-cyclodextrin at 6 h and 24 h (**a**) of incubation. ‘’sEVs mem’’ were depicted with arrowheads. Scale bar 10 μm. Graphs show the total volume of internalized sEVs per cell (**b**), the number of sEV puncta per cell (**c**), the mean volume of internalized sEVs (**d**), the percentage of sEV volume, inside (**e**) or membrane (**f**) per total volume, 6 h and 24 h post-incubation. Colocalization of sEVs with Rab5 (**g**–**i**) and Lamp1 (**j**–**l**) was measured. Scale bar 5 μm. Graphs show colocalization between sEVs and Rab5 (**h**) or Lamp1 (**k**) and the mean volume of colocalized puncta (**i** and **l**, respectively) at different time points, with or without methyl-β-cyclodextrin. Data are presented as the mean ± SEM of minimum three independent cell preparations, with at least two replicates per assay; one-way ANOVA with Tukey’s correction was used for (**d**), (**i**), and (**l**), two-way ANOVA with Tukey’s correction for (**e**), (**f**), (**h**), and (**k**) and multiple t test for (**b**) and (**c**). Statistical significance was set as *p < 0.05, **p < 0.01, ***p < 0.001, ****p < 0.0001

Lipid rafts are defined as micro-domains within the plasma membrane, enriched in cholesterol and sphingolipids, participating in endocytic processes further classified as caveolae- and dynamin-dependent endocytosis, or non-caveolae pathways (dynamin-dependent or independent) [32]. Methyl-β-cyclodextrin is an extensively used pharmacological inhibitor of the pathway, inducing depletion of cholesterol from the plasma membrane [33]. In our experiments, glial cells were incubated with DiI-stained sEVs, with or without methyl-β-cyclodextrin, for 6 h and 24 h. Imaris analysis demonstrated that in microglia, upon methyl-β-cyclodextrin inhibition, sEVs entered cells faster at early time points (Fig. 7a,b), and no difference was detected in the total number of puncta (Fig. 7c) or in the proportions of in/mem sEVs (volume and number of puncta) (Fig. 7e,f, Sup. Figure 7a,b). Regarding the size of internalized sEVs, sEV puncta were larger at early time points, mirroring the size of sEVs of the inside fraction, which seemed to be larger in size at both time points compared to control NT conditions (Fig. 7d, Sup. Figure 7c,d), probably due to the increased internalization rate. Interestingly, sEVs enter faster in methyl-β-cyclodextrin-treated cells even at earlier time points (2 h post-incubation) as compared to NT cells (Sup. Figure 7e). No difference was observed in the proportions of inside/membrane (Sup. Figure 7f). Staining with endosomal markers demonstrated that sEVs reside to a great extent in EE, reaching over 60% colocalization with Rab5 even after 24 h of incubation (Fig. 7g-i), whereas no differences were detected concerning their colocalization with Lamp1 (Fig. 7j-l). The size of endosome-colocalized puncta was similar to that of the NT cells, at both time points.

**Fig. 8:**
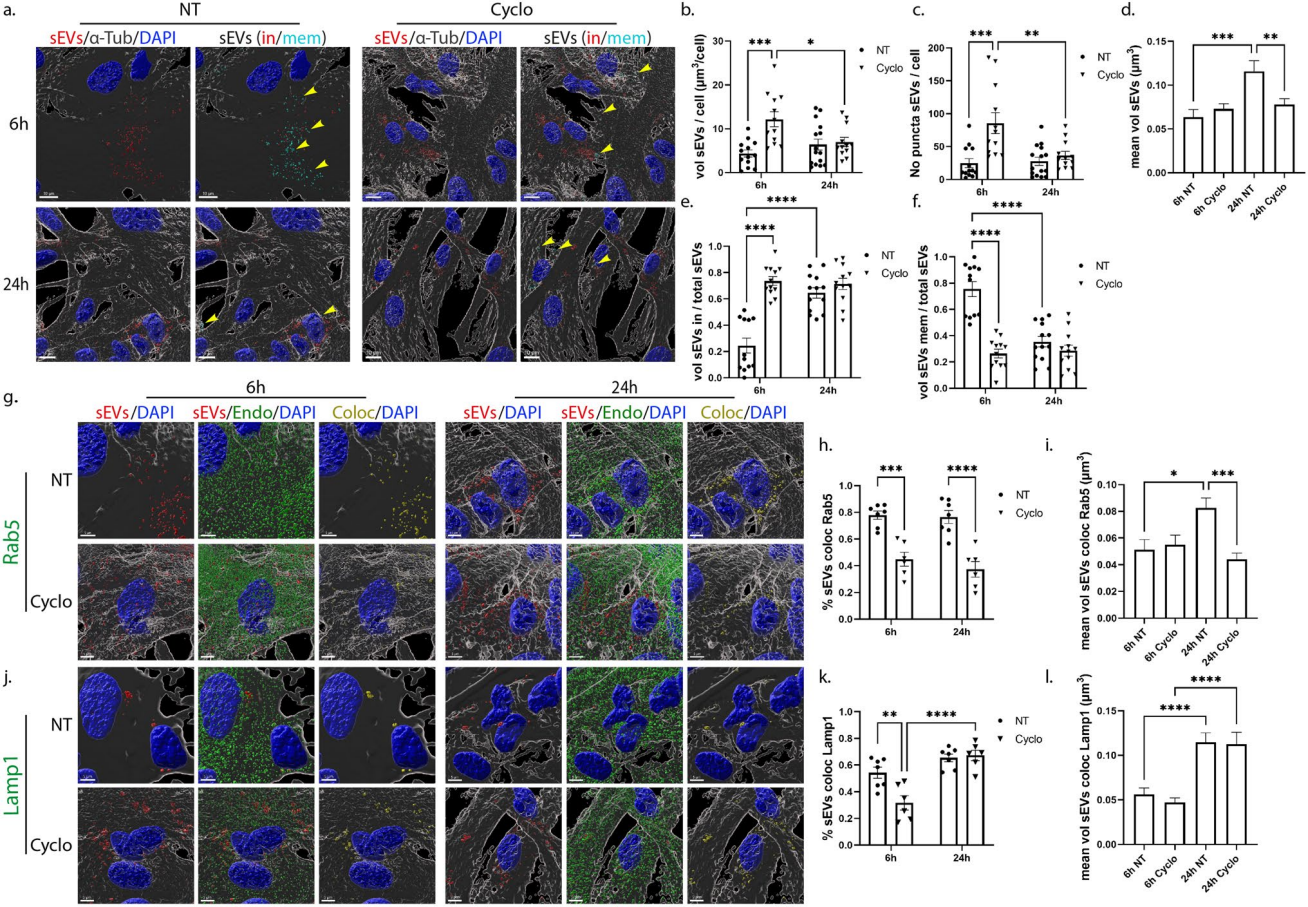
Internalization and endocytic trafficking of sEVs in primary astrocytes upon methyl-β-cyclodextrin treatment. Cells, treated with Dil-labeled sEVs (red), in the absence (NT) or presence of methyl-β-cyclodextrin (Cyclo) for 6 h and 24 h, were fixed and immunostained against α-Tubulin (α-Tub) (gray) and DAPI (blue). Representative Imaris images show the internalization of sEVs and in/mem sEVs without or with methyl-β-cyclodextrin 6 h and 24 h (**a**) post-treatment. ‘’sEVs mem’’ were depicted with arrowheads. Scale bar 10 μm. Graphs present the total volume of internalized sEVs per cell (**b**), the number of sEV puncta per cell (**c**), the mean volume of internalized sEVs (**d**), the percentage of sEV volume, inside (**e**) or membrane (**f**) per total volume, 6 h and 24 h post-incubation. Colocalization of sEVs with Rab5 (**g**–**i**) and Lamp1 (**j**–**l**) was monitored. Scale bar 5 μm. Graphs show colocalization between sEVs and Rab5 (**h**) or Lamp1 (**k**) and the mean volume of colocalized puncta (**i** and **l**) at different time points, with or without methyl-β-cyclodextrin. Data are presented as the mean ± SEM of minimum three independent cell preparations, with at least two replicates per assay; one-way ANOVA with Tukey’s correction was used for (**d**), (**i**) and (**l**), two-way ANOVA with Tukey’s correction for (**b**), (**e**), (**f**), (**h**), and (**k**) and multiple t test for (**c**). Statistical significance was set as *p < 0.05, **p < 0.01, ***p < 0.001, ****p < 0.0001

In primary astrocytic cells, methyl-β-cyclodextrin treatment demonstrated a more prominent effect, with increased total volume and number of puncta 6 h post-addition (Fig. 8a,b,c). Additionally, the proportion of inside sEVs also increased, reaching more than 70%, compared to control NT conditions, where only 20% of sEVs had fully entered the cells (Fig. 8e,f, Sup. Figure 7i,j). Surprisingly, 24 h post-incubation, the total volume and the number of puncta per cell decreased, reaching values similar to the ones observed in NT cells, while the size of sEVs (total, in and mem) remained unaltered over time, in methyl-β-cyclodextrin-treated cells (Fig. 8d, Sup. Figure 7k,l). A number of studies link cholesterol with endosomal flux and endosome fusion and fission dynamics [34, 35]. We noticed that internalized sEVs failed to reside within EE, with almost 40% decrease (Fig. 8g–i) at both time points, upon monitoring the endocytic trafficking in methyl-β-cyclodextrin-treated cell, while Lamp1-positive vesicles were mostly present at later time points (Fig. 8j-l). As documented before [36, 37], methyl-β-cyclodextrin treatment affected both filipin staining and transferrin uptake (Sup. Figure 7g,h,m). Altogether, our data suggest that sEVs enter astrocytes faster when cholesterol is extracted from the plasma membrane.

### The role of actin-dependent endocytic pathway in the internalization and endocytic trafficking of sEVs in primary glia cells

**Fig. 9:**
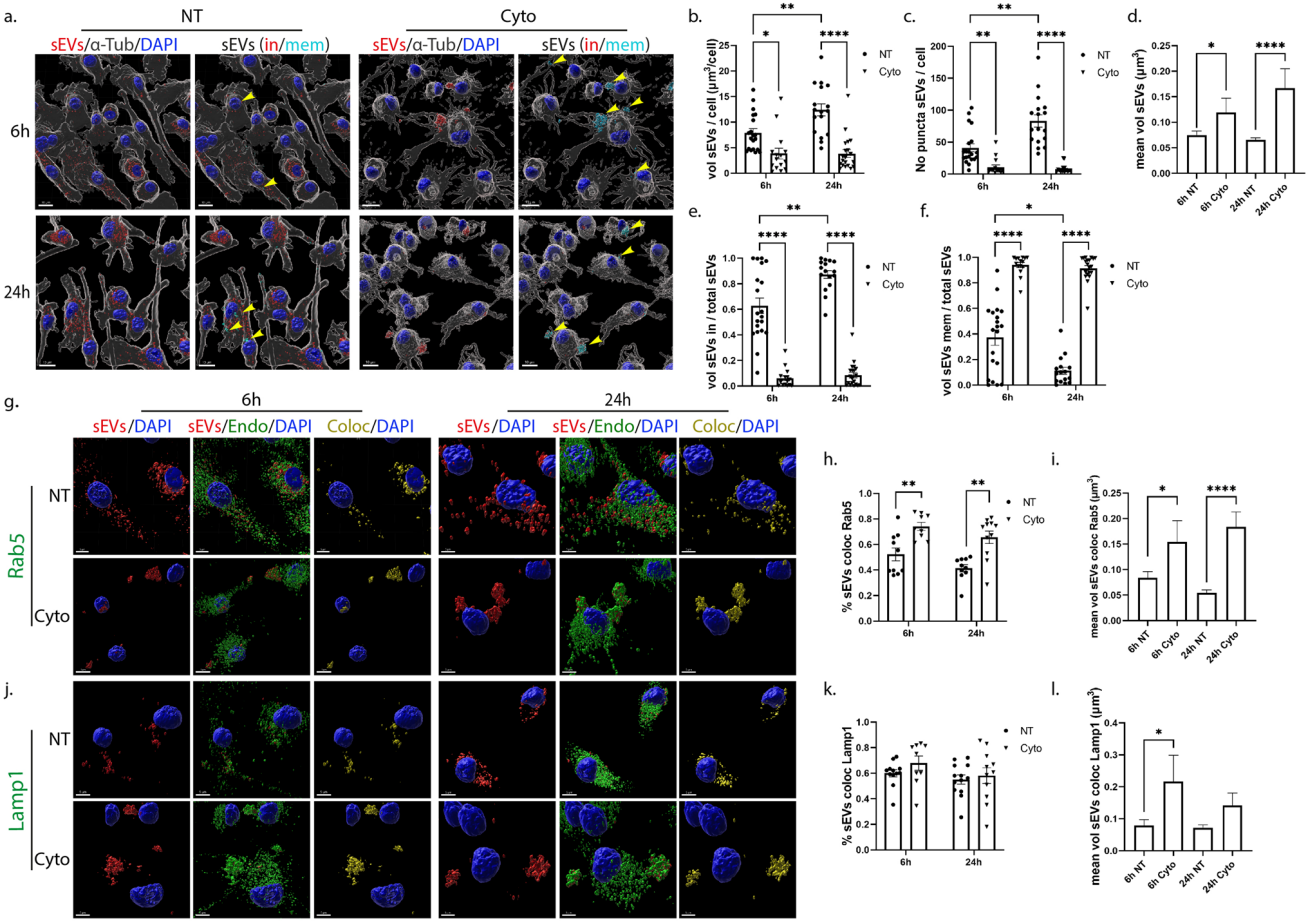
Internalization of sEVs in primary microglia following inhibition of the actin-dependent endocytic pathway. Cells were incubated with Dil-labeled sEVs (red), in the absence or presence of Cytochalasin D (Cyto) for 6 h and 24 h. Cells were fixed and immunostained against α-Tubulin (α-Tub) (gray) and nuclei were stained with DAPI (blue). Representative Imaris images show the internalization of sEVs and in/mem sEVs without or with Cyto, 6 h and 24 h (**a**) post-treatment. ‘’sEVs mem’’ were depicted with arrowheads. Scale bar 10 μm. Graphs present the total volume of internalized sEVs per cell (**b**), the number of sEV puncta per cell (**c**), the mean volume of internalized sEVs (**d**), the percentage of sEV volume, inside (**e**) or membrane (**f**), per total volume, 6 h and 24 h post-incubation. Colocalization of sEVs with Rab5 (**g**–**i**) and Lamp1 (**j**–**l**) was monitored. Scale bar 5 μm. Graphs show colocalization between sEVs and Rab5 (**h**) or Lamp1 (**k**) and the mean volume of colocalized puncta (**i** and **l**, respectively) at different time points, with or without Cyto. Data are presented as the mean ± SEM of minimum three independent cell preparations, with at least two replicates per assay; one-way ANOVA with Tukey’s correction was used for (**d**), (**i**), and (**l**), two-way ANOVA with Tukey’s correction for (**h**) and (**k**) and multiple *t* test for (**b**), (**c**), (**e**), and (**f**). Statistical significance was set as *p < 0.05, **p < 0.01, ***p < 0.001, ****p < 0.0001

To inhibit the pathways of phagocytosis and/or macropinocytosis, we used the pharmacological reagent Cytochalasin D, that disrupts actin network organization, indispensable for both endocytic pathways [12]. DiI-labeled brain-derived sEVs were added on glial cells, in the absence or presence of Cytochalasin D (Cyto), for 6 h and 24 h. Analysis with the Imaris Imaging Software demonstrated compromised sEV entry into microglia cell cytoplasm upon actin-dependent endocytic pathways inhibition as assessed by measurements of total volume and number of puncta per cell as well as from the proportions of the in/mem fractions (volume, number of puncta) (Fig. 9a,b,c,e,f, Sup. Figure 8a,b). Upon Cyto-treatment, over 90% of sEVs accumulated on the plasma membrane at both time points, creating large puncta unable to enter cells (Fig. 9d,e,f, Sup. Figure 8c,d). Further, treatment with 5-(N-ethyl-N-isopropyl) amiloride (EIPA), which blocks macropinocytosis and to a lesser extent phagocytosis, prevented sEV entry, 6 h post-treatment (Sup. Figure 8e). Analysis of the endocytic trafficking showed increased colocalization with Rab5 in Cyto-treated cells, 70–80%, resulting in larger colocalized puncta at both time points (Fig. 9g–i). Additionally, sEVs that remain on the plasma membrane were Lamp1-positive in Cyto-treated cells after 6 h and 24 h of incubation, and their size increased 6 h post-incubation (Fig. 9j–l).

**Fig. 10:**
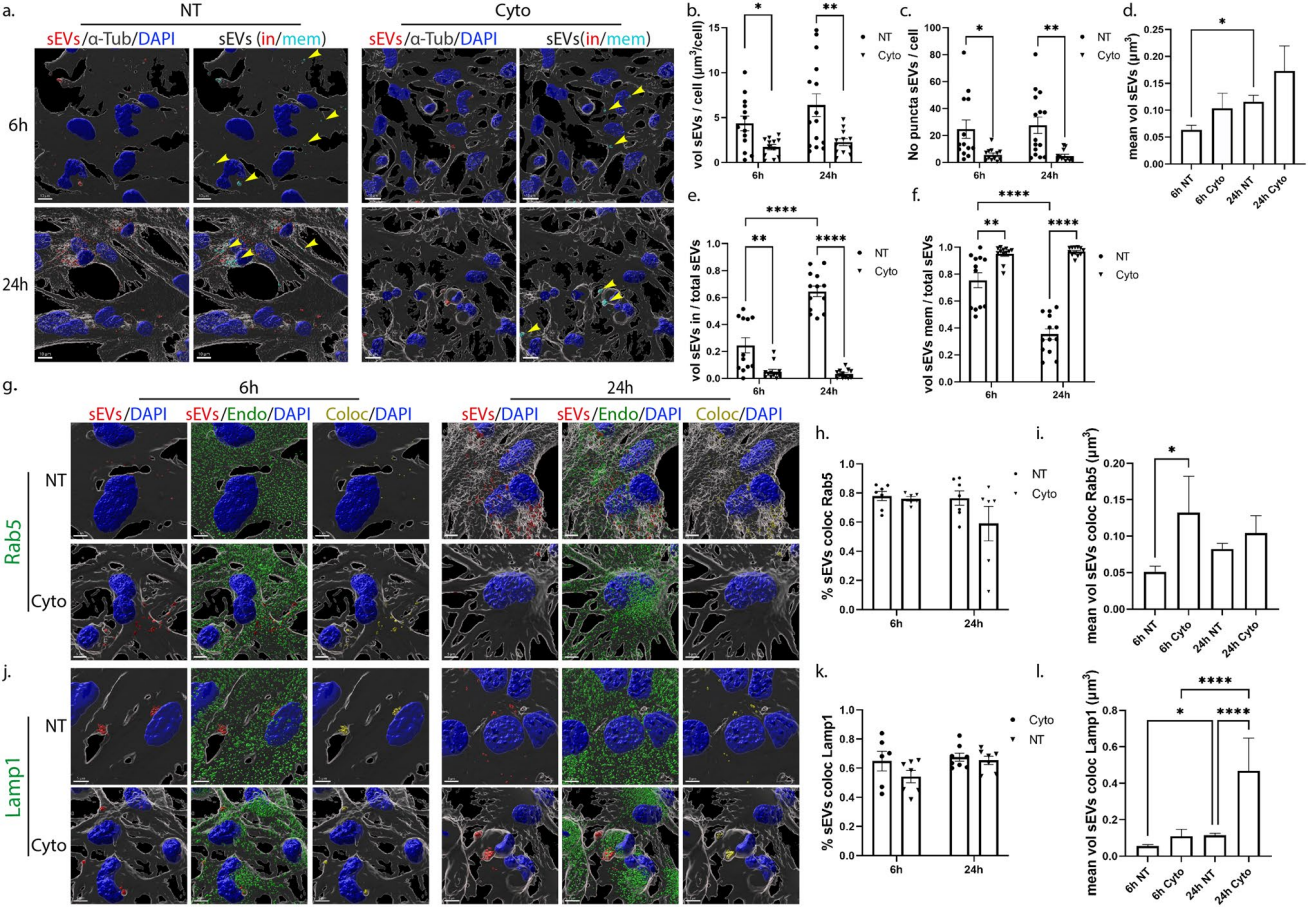
Uptake of sEVs in primary astrocytes through the actin-dependent endocytic pathway. Cells, incubated with Dil-labeled sEVs (red), in the absence or presence of Cytochalasin D (Cyto) for 6 h and 24 h, were fixed and immunolabeled against α-Tubulin (α-Tub) (gray) and nuclei were stained with DAPI (blue). Representative Imaris images show the internalization of sEVs and in/mem sEVs without or with Cyto, 6 h and 24 h (**a**) post-treatment. ‘’sEVs mem’’ was depicted with arrowheads. Scale bar 10 μm. Graphs present the total volume of internalized sEVs per cell (**b**), the number of sEV puncta per cell (**c**), the mean volume of internalized sEVs (**d**), the percentage of sEV volume, inside (**e**) or membrane (**f**) per total volume, 6 h and 24 h post-incubation. Colocalization of sEVs with Rab5 (**g**–**i**) and Lamp1 (**j**–**l**) was evaluated. Scale bar 5 μm. Graphs show colocalization between sEVs and Rab5 (**h**) or Lamp1 (**k**) and the mean volume of colocalized puncta (**i** and **l**, respectively) at different time points, with or without Cyto. Data are presented as the mean ± SEM of minimum three independent cell preparations, with at least two replicates per assay; one-way ANOVA with Tukey’s correction was used for (**d**), (**i**) and (**l**), two-way ANOVA with Tukey’s correction for (**e**), (**f**), (**h**), and (**k**) and multiple t test for (**b**) and (c). Statistical significance was set as *p < 0.05, **p < 0.01, ***p < .001, ****p < 0.0001

Likewise, in astrocytes, inhibition of the actin-dependent pathways prevented sEV entry with over 90% remaining on the plasma membrane at both time points (Fig. 10a,b,c,e,f, Sup. Figure 8g,h). Even though the size of total puncta did not change upon Cyto-treatment, the differences in puncta size of the mem fraction between the treatments were more prominent at 24 h post-incubation. Such difference was not observed at 6 h of incubation, as sEVs still resided on the plasma membrane in NT cells (Fig. 10d, Sup. Figure 8i,j). Treatment with EIPA restricted sEVs from entering astrocytes at 6 h and 24 h of incubation (Sup. Figure 8k). Even though sEVs were restricted to the plasma membrane upon Cyto application, the components of the endocytic trafficking (Rab5 and Lamp1) were found colocalized with them on the plasma membrane (Fig. 10g,h,j,k), possibly in an attempt to process those that are blocked from entering cells. Changes in the size of Rab5-colocalized puncta were detected 6 h post-incubation and of Lamp1-colocalized puncta after 24 h (Fig. 10i,l). Cyto-treatment, but not Dyn, disrupted the actin network organization in both cell types, as depicted by fluorescent phalloidin that stains F-actin and is commonly used to assess actin cytoskeleton (Sup. Figure 8f,l). Altogether, the actin-dependent endocytic pathways, macropinocytosis and/or phagocytosis, seemed to be responsible for the internalization of sEVs in both glial cell types.

To assess the endolysosomal maturation under the different conditions, we measured Rab5 and Lamp1 total volume per cell, as well as the colocalization between them (Sup. Figure 9). Upon Dyn treatment, Rab5 volume increased in both cell types and Lamp1 decreased only in microglia. Upon methyl-β-cyclodextrin treatment, Rab5 volume increased and Lamp1 decreased in microglia; however, in astrocytes they remained unchanged. Cyto affected Rab5 total volume only in astrocytes and Lamp1 only in microglia (Sup. Figure 9a,b,e,f). The percentage of Rab5 colocalization with Lamp1 was poor in all conditions, in both cell types (Sup. Figure 9c,g). Lamp1 overlap with Rab5 remained low and unaltered in dyn and cyto-treated cells with a slight increase in methyl-β-cyclodextrin-treated cells in microglia, and in cyto-treated cells in astrocytes (Sup. Figure 9d,h). These data designate that Dyn affects endolysosomal maturation as described before [38], in both microglia and astrocytes, and methyl-β-cyclodextrin affects endosomal flux only in microglia, not in astrocytes. Cyto-treatment increased Rab5 in astrocytes and decreased Lamp1 in microglia. Altogether, it seems that endolysosomal maturation is differentially affected in the different cell types.

### sEV-mediated transmission of fibrillar α-synuclein in primary microglia cells

**Fig. 11:**
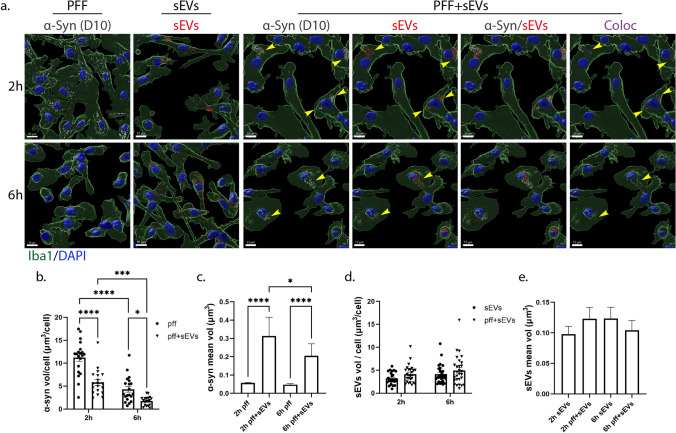
sEV-dependent α-Syn transmission in primary microglia. a. α-Syn pre-formed fibrils (PFF) were pre-incubated with sEVs derived from SNCA KO mouse brains (PFF + sEVs), for 20 h at 37 ^o^C. Microglia cells were treated with PFFs or sEVs alone or PFF + sEVs for 2 h. Uptake of α-Syn and sEVs were monitored 2 h and 6 h post-addition. Cells were fixed and immunostained against α-Syn (D10, light gray) and Iba1 (green). Cell nuclei were stained with DAPI (blue). Representative Imaris images show the uptake of α-Syn and sEVs in all three conditions (PFFs, sEVs, and PFF + sEVs) 2 h and 6 h post-treatment. Arrowheads depict PFF colocalization with sEVs. Colocalization of α-Syn with sEVs is depicted in magenta. Scale bar 10 μm. Graphs present the total volume of α-Syn per cell (**b**), the mean volume of α-Syn individual puncta (**c**), the total volume of sEVs per cell (**d**), and the mean volume of sEV puncta (**e**) under the different conditions. Data are presented as the mean ± SEM of minimum three independent cell preparations, with at least two replicates per assay; one-way ANOVA with Tukey’s correction was used for (**c**) and (**e**), two-way ANOVA with Tukey’s correction for (**d**), and multiple t test for (**b**). Statistical significance was set as *p < 0.05, **p < 0.01, ***p < 0.001, ****p < 0.0001

To elucidate the role of sEVs in the transmission and clearance of pathogenic protein aggregates, we used structurally well-defined human recombinant α-Syn fibrils (PFFs, pre-formed fibrils) that seed endogenous α-Syn after take up in neuronal cells [22, 25] and monitored how sEV association affected their internalization and clearance pathways in microglia. Recent studies have reported that sEVs associate with fibrillar α-Syn and A*β* and either block α-Syn internalization in primary neuronal cells [20] or enhance A*β* clearance by microglia [39]. We pre-incubated sEVs and α-Syn-PFFs for 20 h and performed sucrose gradient ultracentrifugation, followed by immunoblotting against α-Syn with the specific antibody D10 that recognizes the human α-Syn C-terminal end. α-Syn alone is detected in the '’f’’ fraction, whereas pre-incubated α-Syn with sEVs is detected in the ‘’c’’ and more prominently in the ‘’d’’ fraction, denoting α-Syn association with sEVs (Sup. Figure 10a). Next, we exposed microglia to the sEV-associated PFFs for 2 h. α-Syn internalization and intracellular trafficking were assessed 2 h and 6 h post-addition. Cultures treated with α-Syn-PFFs or sEVs alone were used as controls. Staining against α-Syn, followed by confocal microscopy and Imaris analysis, revealed that α-Syn-PFFs entered microglia cells either alone or in association with sEVs (PFF + sEV). PFFs alone were taken up more efficiently than PFF + sEV by microglia, 2 h post-incubation (Fig. 11a,b). Interestingly, α-Syn-PFFs were cleared from the cells, whether associated or not to sEVs, 6 h post-addition (Fig. 11a,b). The clearance rate of PFFs was slightly but not significantly faster (2.614 for PFFs and 3.306 for PFFs + sEV) when they were associated with sEVs (Sup. Figure 10b). Additionally, the size of α-Syn puncta observed inside cells was considerably higher when cells were incubated with sEV-associated PFFs (Fig. 11c). 24 h post-addition, α-Syn-PFFs were completely cleared from the cells under all experimental conditions (PFF and PFF + sEVs; data not shown). sEV association with PFFs did not affect the internalization of sEVs by microglia nor did it affect their size (Fig. 11a,d,e).

**Fig. 12:**
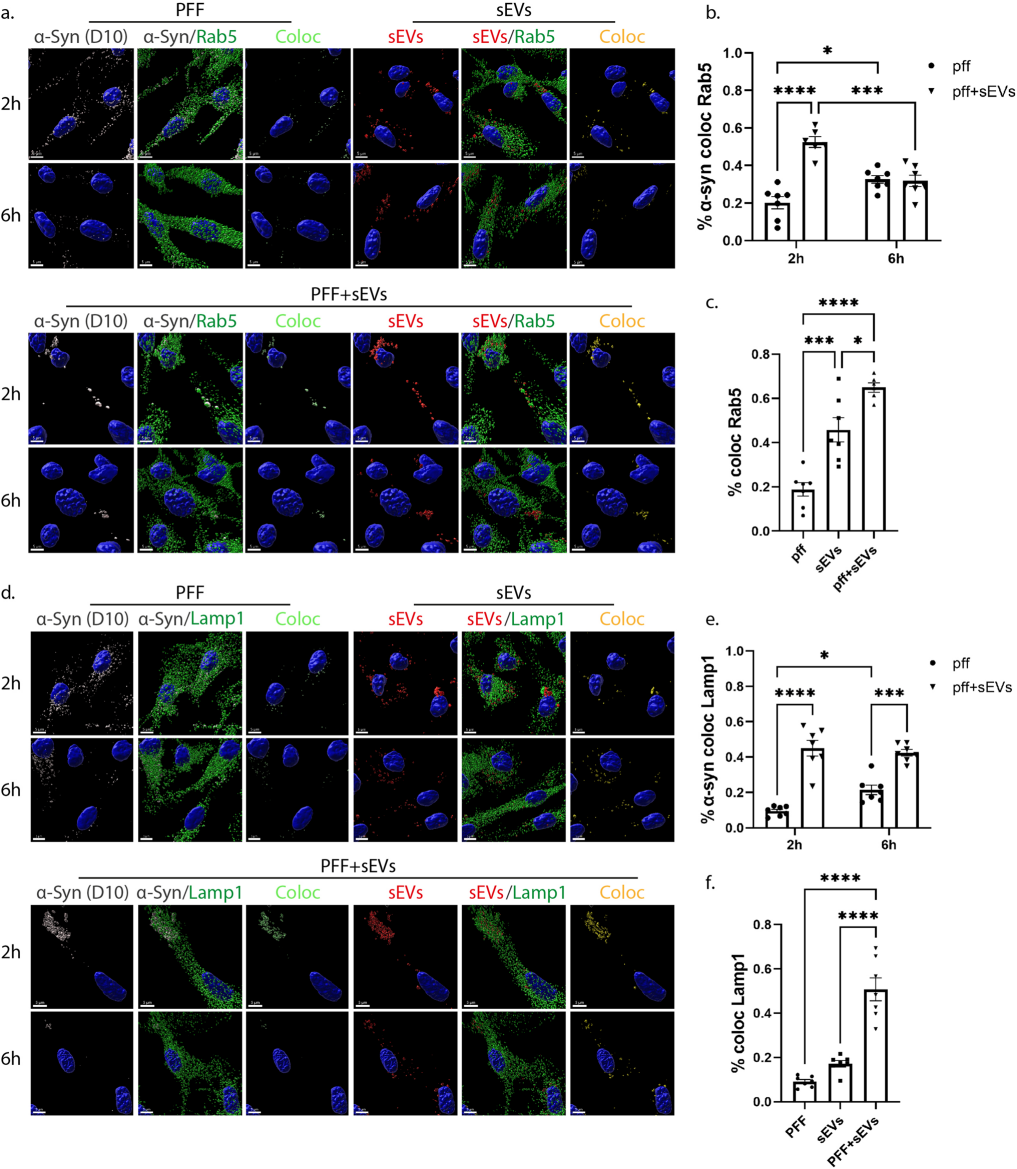
sEV-dependent α-Syn intracellular trafficking in primary microglia. α-Syn pre-formed fibrils (PFF) were pre-incubated with sEVs derived from SNCA KO mouse brains (PFF + sEVs), for 20 h at 37 ^o^C. Microglia cells were treated with PFFs or sEVs alone or PFF + sEVs for 2 h. Intracellular trafficking of α-Syn and sEVs were monitored 2 h and 6 h post-addition. The endocytic trafficking was monitored by measuring colocalization of α-Syn (depicted in light green) and sEVs (depicted in yellow) with Rab5 (**a**–**c**) and Lamp1 (**d**–**f**), at the different treatments (PFFs, sEVs and PFF + sEVs). Scale bar 5 μm. Graphs show colocalization of α-Syn with Rab5 (**b**) and Lamp1 (**e**) in the different conditions (PFFs, sEVs, PFF + sEVs), as well as colocalization of α-Syn/sEV colocalized puncta with Rab5 (**c**) and Lamp1(**f**), in the PFF + sEVs compared to the PFF or sEVs alone treatments, 2 h post-incubation. Data are presented as the mean ± SEM of minimum three independent cell preparations, with at least two replicates per assay; one-way ANOVA with Tukey’s correction was used for (**c**) and (**f**), and two-way ANOVA with Tukey’s correction for (**b**) and (**e**). Statistical significance was set as *p < 0.05, **p < 0.01, ***p < 0.001, ****p < .0001

To further investigate the pathway followed post-internalization, we stained with Rab5 and Lamp1, and following colocalization analysis with the Imaris Imaging software, we found that PFFs colocalized with both endocytic markers. However, colocalization was substantially higher in the presence of sEVs (Fig. 12, Sup. Figure 10). More specifically, in PFF-treated cells, the percentage of colocalization with Rab5 and Lamp1 was 20% and 8%, respectively, after 2 h, increasing to 33% and 20%, at 6 h post-PFF addition. In PFF + sEV-treated cells, colocalization with both Rab5 and Lamp1 was higher (53% and 45%, respectively) after 2 h of incubation, compared to PFF-alone treated cells. After 6 h of treatment, colocalization with Rab5 dropped to 30%, while with Lamp1, it remained at the same levels (Fig. 12a,b,d,e). Colocalization of sEVs with Rab5 did not change following association with PFFs; on the contrary, colocalization of sEVs with Lamp1 was higher when they were associated with PFFs (Fig. 12a,d, Sup. Figure 10c,d). Furthermore, α-Syn/sEV colocalized puncta displayed even higher colocalization with both Rab5 and Lamp1 (64% and 50%, respectively) when compared to cells treated with PFF- or sEV-alone (Fig. 12c,f). Our data indicate that in the presence of sEVs, fibrillar α-Syn is targeted to the endolysosomal pathway for subsequent degradation, suggesting a critical role of sEVs in the clearance process of α-Syn aggregates in microglia cells.

## Discussion

**Fig. 13:**
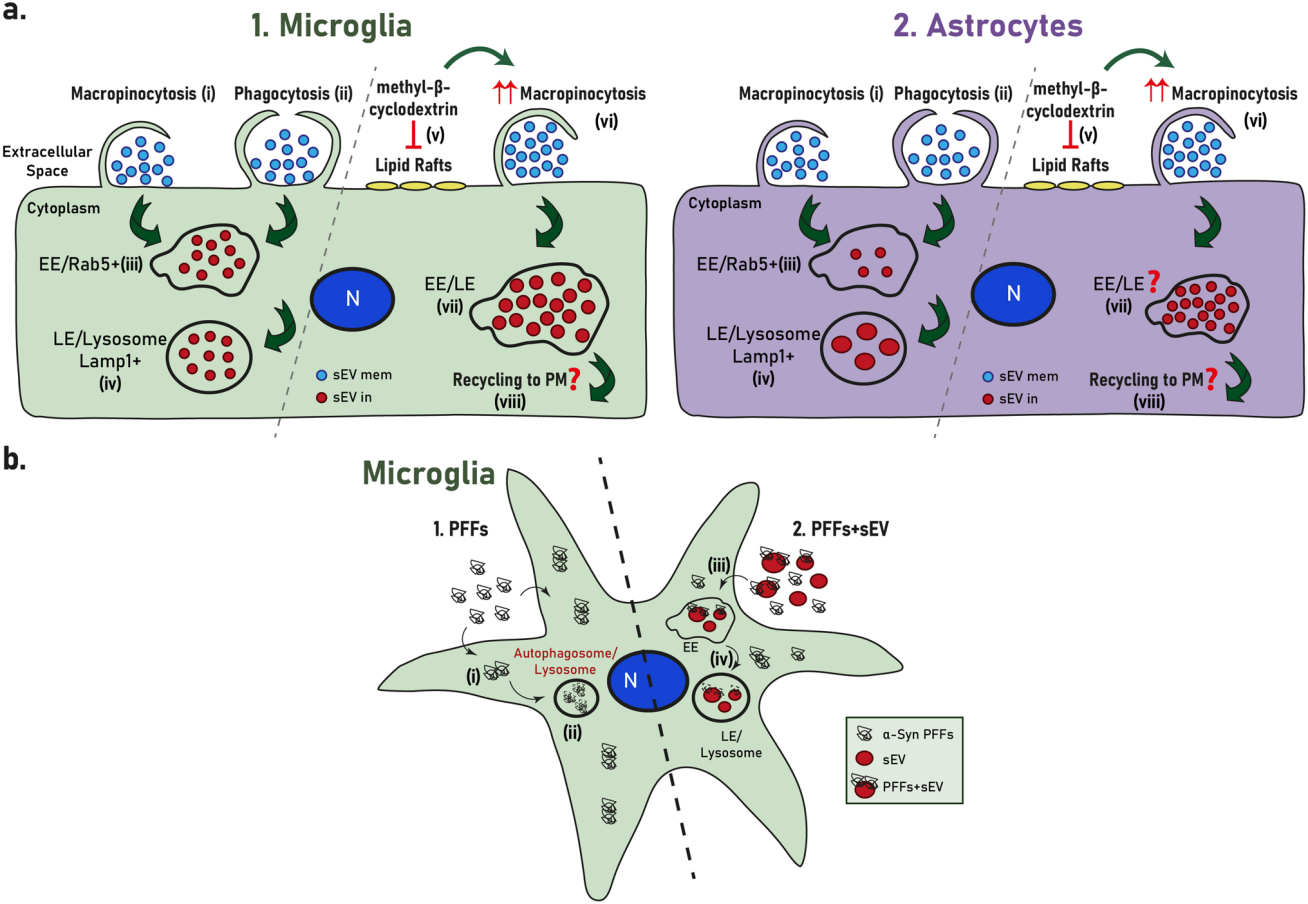
**a** Internalization processes and endocytic trafficking of brain-derived sEVs in microglia (1) and astrocytes (2). Brain-derived sEVs are taken up by both glial cell types; however, microglia demonstrate a more efficient internalization rate compared to astrocytes. Both cell types utilize the actin-dependent pathways, macropinocytosis (i) and/or phagocytosis (ii), for the transfer of sEVs within the cells and subsequently target them to the endolysosomal pathway (iii, iv) for further processing. Cholesterol depletion (v) induces sEV internalization in both microglia and astrocytes, possibly through induction of macropinocytosis (vi), exhibiting though a differential impact on sEV endosomal trafficking between the two cell types (vii). Cholesterol’s contribution to the interplay between endosomal recycling to the plasma membrane and/or lysosomal sorting requires further investigation (viii). **b**. sEV-dependent α-Syn transmission in microglia. Fibrillar α-Syn-associated sEVs (PFFs + sEVs) (2) are internalized (iii), enter the endosomal pathway (EE and LE), and are targeted to the lysosome (iv) for subsequent degradation. In the absence of sEVs (1), α-Syn-PFFs fail to enter the endosomal pathway, accumulate in the cytoplasm (i), and are cleared from the cells, possibly through autophagy (ii) [79]

In the present study, we explored the internalization processes and endocytic trafficking of brain-derived sEVs in primary microglia and astrocytes. Our data indicate that brain-derived sEVs are taken up by both glial cell types, with higher internalization rate for microglia (Figs. 1, 11,13a). Both cell types utilize the actin-dependent pathways, macropinocytosis and/or phagocytosis, for the transfer of sEVs within the cells (Figs. 9, 10, 13a) and subsequently target them to the endolysosomal pathway for further processing (Figs. 3, 4, 13a). Additionally, we assessed the role of lipid rafts in sEV uptake (Figs. 5–8) and interestingly found that sEVs are internalized faster when cholesterol is stripped off the plasma membrane in both microglia and astrocytes (Figs. 7,8,13a). Finally, we investigated the potency of sEVs in sequestering pathogenic proteins, present in the extracellular space, into microglia using α-syn PFFs as a paradigm. We showed that fibrillar α-Syn species when associated with sEVs enter the endosomal pathway and are targeted to the lysosome for subsequent degradation. In the absence of sEVs, only a small portion of fibrillar α-Syn is sorted to the endolysosomal pathway (Figs. 11, 12, 13b). sEVs mediate communication between neuron and glial cells, hence participating in various functions of the brain, both in health and disease [40]. Thus far, studies have focused mostly on glia-derived sEVs and their role in neuronal function. Limited studies have addressed sEV internalization in glial cells; however, strong evidence indicates that, depending on the disease (i.e., brain tumor, ischemic stroke, autism, and neurodegenerative diseases), sEV uptake can be either beneficial or detrimental [41–48]. Our data suggest that microglia possess mechanisms for rapid response to extracellular changes for targeted degradation and the subsequent inflammatory response, whereas astrocytes slowly endocytose sEVs protecting the cell from unrestrained signaling response, reflecting the cells’ key functions in the CNS. Noteworthy, our experimental approach entails limitations regarding the cell origin of sEVs derived from whole brain extracts.

Over the last decade, numerous reports have aimed to elucidate the internalization and endocytic trafficking of sEVs, and hence the pathways essential for cell-to-cell communication. Clathrin-mediated endocytosis is the best molecularly defined pathway involved in the uptake of sEVs by numerous cells [49–53]. In several cases, it was reported that more than one pathways may independently and cooperatively contribute to the endocytic processes [12, 54–57]. Additionally, identifying the endocytic pathway involved remains a challenge due to the cross-reactive effect of pharmacological inhibitors [33, 58]. In this regard, dynasore is used to inhibit dynamin function, implicated in vesicle fission from the plasma membrane after internalization through the clathrin- or caveolin-mediated endocytosis, but also reduces cholesterol on the plasma membrane, thus altering lipid composition in lipid rafts in a dynamin-independent manner [59]. In our experiments, dynasore treatment had no effect on sEV uptake by primary microglia cells (Fig. 5, Sup. Figure 6); however, it increased their internalization in astrocytes, as most sEVs were found to localize within the cytoplasm with a small proportion remaining on the plasma membrane (Fig. 6, Sup. Figure 6). Several studies have demonstrated the essential role of lipid rafts in sEV uptake by cancer cells, T cells, and HUVECs [54, 60–62]. On the contrary, in PC12 cells, sEV uptake was increased upon methyl-*β*-cyclodextrin inhibition [57]. Accordingly, in our experiments, methyl-*β*-cyclodextrin treatment appeared to induce sEV internalization in both cell types (Figs. 7, 8, Sup. Figure 7), whereas dynasore increased uptake only in astrocytes, probably because of its effect on cholesterol distribution. Taken together, sEV uptake is negatively regulated by cholesterol in both microglia and astrocytes, possibly due to alterations in plasma membrane fluidity and tension, accelerating uptake by other pathways. Furthermore, it has been reported that methyl-*β*-cyclodextrin may facilitate the secretion of the vesicular content [63], hence justifying the decrease of sEVs observed 24 h post-incubation in astrocytes. In microglia, considering the fast internalization processes observed, no effect was detected with methyl-β-cyclodextrin. Of note, cholesterol levels are decreased in AD patients, mostly in brain areas more susceptible to AD (cortex and hippocampus) [64]. Additionally, TREM2, the transmembrane receptor in microglia implicated in late-onset AD, regulates cholesterol and lipid metabolism [65]. Considering the imbalance between endosomal/autophagic degradation and secretion of toxic protein species in AD, due to the impairment of lysosomal degradation [66], our data could yield interesting information concerning sEV-mediated A*β*/Tau transmission, microglia activation, and disease progression in AD.

Macropinocytosis and/or phagocytosis are implicated in sEV uptake by different cell types, including professional and non-professional phagocytes, resulting in the formation of vesicles, macropinosomes, and phagosomes, respectively, with size larger than 0.2 μm [13, 67–71]. Cytochalasin D is a widely used pharmacological inhibitor that blocks both pathways, through interference with actin dynamics. Other pharmacological reagents (wortmannin and LY294002) target components shared by both pathways, and while amiloride or its analogs (i.e., EIPA) seemingly inhibit macropinocytosis with high specificity, it may affect actin as well [33, 72]. In the present study, Cytochalasin D and EIPA treatment perturbed internalization of sEVs in both microglia and astrocytes, leading to the conclusion that macropinocytosis and/or phagocytosis are the main endocytic pathway(s) for sEV uptake in glial cells (Figs. 9,10, Sup. Figure 8). It has been recently shown that lipid raft disruption through methyl-*β*-cyclodextrin treatment induces the activation of phospholipase D2 (PLD2) which in turn activates macropinocytosis [73, 74], leading to the faster internalization observed in both glial cell types upon cholesterol depletion. This finding, together with the observed EIPA inhibition results further implicates macropinocytosis in sEV uptake. Our results are in agreement with past studies where oligodendroglia cell-derived sEVs are restricted from entering microglia upon amiloride treatment [13].

Thus far, limited studies have addressed the post-internalization fate of sEVs and subsequent endosomal trafficking [18, 67, 75]. Here, the differences observed in the proportion of sEVs residing in EE and LE/Lysosomes as well as in the size of colocalized sEVs between the cell types at different time points support faster internalization processes and subcellular trafficking to lysosomes in microglia (Figs. 3,4, Sup. Figure 4, 5). Astrocytes exhibit a pronounced localization of sEVs at EE at early time points, as internalization is gradual, with most sEVs residing on the plasma membrane. In agreement with the recent reports which detect Lamp1 in EE-positive compartments [76, 77], our data also show that sEVs residing on the plasma membrane are Lamp1-positive (Figs. 4, 9, 10). The role of Lamp1 in the plasma membrane remains elusive and further investigation linking its function to EE, LE, or lysosomes is required. Interestingly, and in accordance with previous studies [38, 59], dynasore seems to interfere with endosomal maturation and trafficking (Sup. Figure 9), resulting in sEV puncta restricted from sorting to the endolysosomal pathway (Figs. 5,6, Sup. Figure 6, 9). Cholesterol depletion is proposed to affect endosomal flux and LE maturation [34, 35], though it appeared to have a differential impact on sEV trafficking between the two cell types. The distinct localization of sEVs in the endosomal compartments upon methyl-*β*-cyclodextrin treatment in microglia and astrocytes (Figs. 7,8), probably reflect the differential effect of methyl-β-cyclodextrin on the endolysosomal maturation and secretion [63] in the two cell types (Sup. Figure 9). Future investigation is needed to address the interplay between endosomal recycling to the plasma membrane and lysosomal sorting, elucidating cholesterol’s contribution to the distinct pathways and thus to the fate of sEV cargo.

To investigate the role of sEVs in the clearance of pathogenic protein aggregates by microglia, we used fibrillar α-Syn. It has been shown that microglia exposure to PFFs induces the release of α-Syn-containing sEVs and pro-inflammatory cytokines, further contributing to the progression of α-Syn pathology in neurons [78]. Our data demonstrate that fibrillar α-Syn largely fails to enter the endosomal pathway, accumulates in the cytoplasm and is cleared from the cells possibly through autophagy, as previously described [79]. sEVs significantly facilitate the process of sorting of α-Syn-PFFs to the endosomal pathway for rapid degradation by the lysosome (Figs. 11, 12,13b), as previously shown in an AD model where A*β*-associated sEVs were sorted to lysosomes for subsequent degradation in microglia [39]. In addition, a recent report has proposed that fibrillar α-Syn is attached on the membrane of intraluminal vesicles within multivesicular bodies thus facilitating its transport on the surface of sEVs [80], attributing further significance to the scavenging properties of sEVs described in this study. Collectively, it appears that association of fibrillar α-Syn with sEVs regulates the subsequent clearance pathway, thus modulating degradation efficiency and likely the following inflammatory response. Although it has been suggested that the lysosome is the final destination for sEVs [81–83], recent reports support the idea that their cargo can escape endosomes and be targeted to the cytoplasm where they can exert their function [18, 19, 84, 85]. This is a proposed mechanism through which sEV-delivered pathogenic proteins escape lysosomal degradation through vesicular rupture and initiate pathogenic processes in the cytoplasm. Thus, to further examine the role of sEVs in α-Syn transmission and propagation, it would be interesting to investigate fibrillar α-Syn-induced vesicular rupture and/or sEV release in our model, as well as the effect on microglia activation and inflammatory response, and in turn transmission and disease progression in neurons.

For our study, we utilized confocal imaging followed by deconvolution and through 3D reconstruction with the Imaris Imaging software, we thoroughly analyzed sEV internalization and endosomal sorting using a variety of parameters, rigorously dissecting the pathways involved. To the best of our knowledge, this is the first report that characterizes the endocytic processes implicated in sEV uptake by astrocytes. Noteworthy, we provide information on the endosomal sorting pathways implicated in sEV subcellular trafficking in both glial cell types, a field yet understudied in the CNS, and ultimately designate the differences between the cell types, hence the distinct responses of glial cells in processing sEVs and their cargo, possibly reflecting their contribution in the functioning of the glia-neuron synapse. Nonetheless, future investigation is required to study the correlation of sEV origin and recipient cell specificity, mainly in astrocytes where uptake appears to be regulated. Furthermore, our results suggest that manipulation of endocytic pathways and intracellular trafficking to endo-lysosomes may allow clearing sEV-mediated pathogenic protein aggregates transmission and controlling the associated inflammatory responses. Collectively, our study delineates important pathways involved in sEV trafficking in the CNS, paving the way for a better understanding of cell-to-cell communication in health and disease.

## Data availability statement

The data that support the findings of this study are available from the corresponding author upon reasonable request.

## Supplementary Information

Below is the link to the electronic supplementary material.

Supplementary file1 (XLSX 2317 KB)

Supplementary file2 (DOCX 15162 KB)

## Abbreviations

AD: Alzheimer's disease
ALS: Amyotrophic lateral sclerosis
α-Tub: α-Tubulin
α-Syn: α-Synuclein
BBB: Blood–brain barrier
CDE: Caveolin-dependent endocytosis
CME: Clathrin-mediated endocytosis
CNS: Central nervous system
Cyclo: Methyl-β-cyclodextrin
Cyto: Cytochalasin D
Dyn: Dynasore
EE: Early endosomes
EM: Electron microscopy
LE: Late endosomes
NTA: Nanoparticle tracking analysis
PD: Parkinson’s disease
PFF: Pre-formed fibrils
sEVs: Small extracellular vesicles
sEVs in: SEVs inside
sEVs mem: SEVs membrane
