# Supplementary material for: Differential intracellular trafficking of extracellular vesicles in microglia and astrocytes
Source: Cell Mol Life Sci. 2023 Jun 30;80(7):193. doi: 10.1007/s00018-023-04841-5 (PMC10313565; doi:10.1007/s00018-023-04841-5)
Supplement: Supplementary file 2 — Supplementary file2 (DOCX 15162 KB) [file 18_2023_4841_MOESM2_ESM.docx]

**Differential intracellular trafficking of extracellular vesicles in microglia and astrocytes**

**Running title:** Uptake of ­**extracellular vesicles** by glial cells

Marina Pantazopoulou^1^, Agaristi Lamprokostopoulou^2#^, Dimitra Sotiria Karampela^2#^, Anastasia Alexaki^2^, Anastasios Delis^2^, Audrey Coens^3^, Martina Samiotaki^4^, Anastasios G. Kriebardis^5^, Ronald Melki^3^, Stamatis N. Pagakis^2^, Leonidas Stefanis^1^, Kostas Vekrellis^2^

^1^Biomedical Research Foundation Academy of Athens- BRFAA, Clinical- Experimental Surgery & Translational Research, Athens, Greece

^2^Biomedical Research Foundation Academy of Athens- BRFAA, Centre of Basic Research, Athens, Greece

^3^Institut Francois Jacob (MIRCen), CEA and Laboratory of Neurodegenerative Diseases, CNRS, Fontenay-Aux-Roses cedex, France

^4^Institute for bio-innovation, Biomedical Sciences Research Center 'Alexander Fleming', Fleming 34, Vari, 16672, Greece.

^5^Laboratory of Reliability and Quality Control in Laboratory Hematology (HemQcR), Department of Biomedical Sciences, School of Health & Welfare Sciences, University of West Attica (UniWA), Egaleo, Greece

# These authors contributed equally to this work

**^*^Correspondence to:**

Dr Marina Pantazopoulou

Biomedical Research Foundation of the Academy of Athens

4, Soranou tou Efesiou Street

Athens, Greece 11527

Email: mpantazopoulou@bioacademy.gr

ORCID ID: https://orcid.org/0000-0001-8262-6274

**Supplementary Information**

_­­­
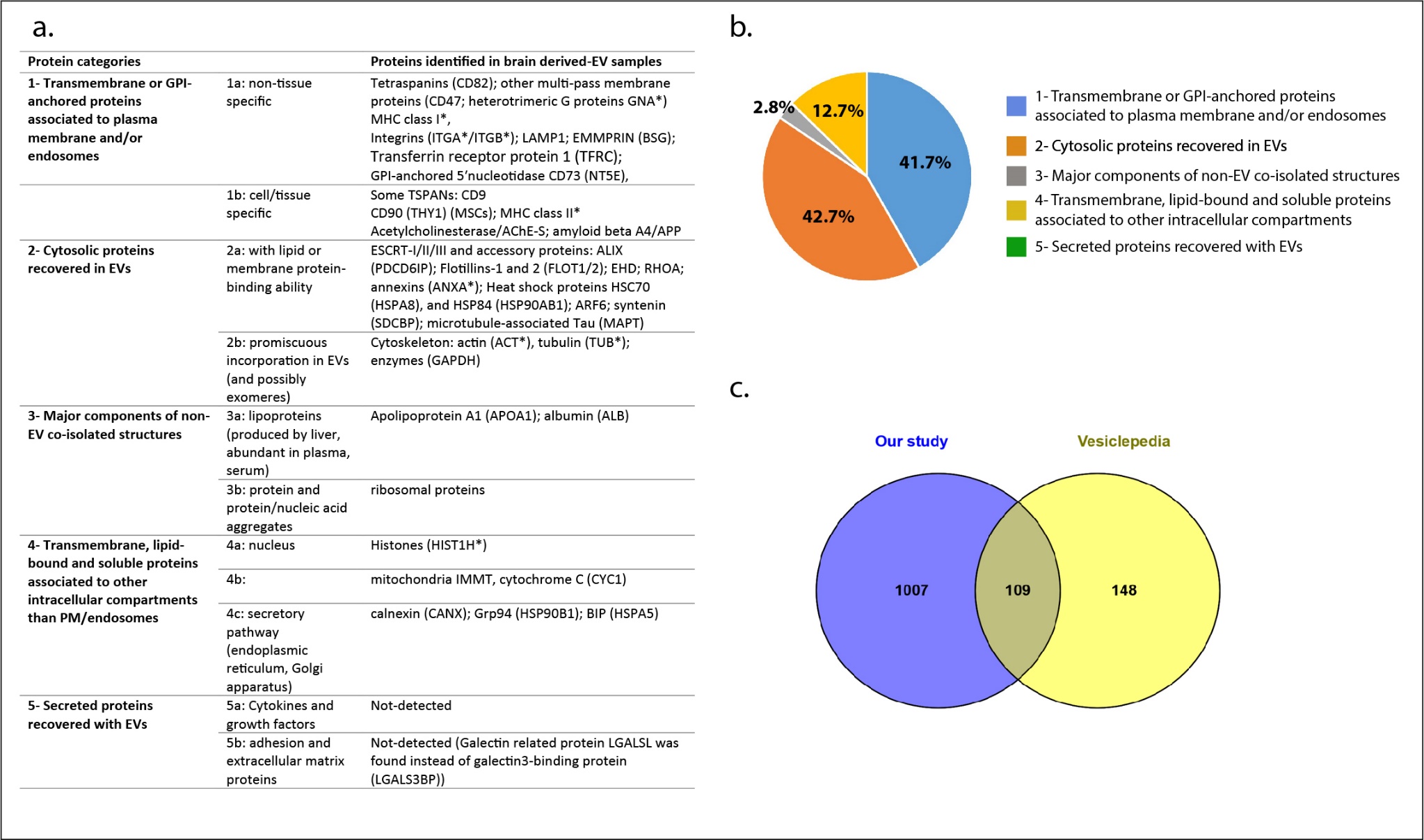
_

**Sup. Fig. 1. Proteomic analysis of mouse brain-derived EVs.** Protein categories described in Théry *et al.*, 2018 (*: used for families of multiple proteins) (a) and proportions represented in each protein category (b). Venn diagram comparing the proteins present in brain-derived EVs in our study with those in Vesiclepedia. 109 of the top 148 proteins encountered in EVs, according to Vesiclepedia, were identified in the EV-enriched fraction (c).

**
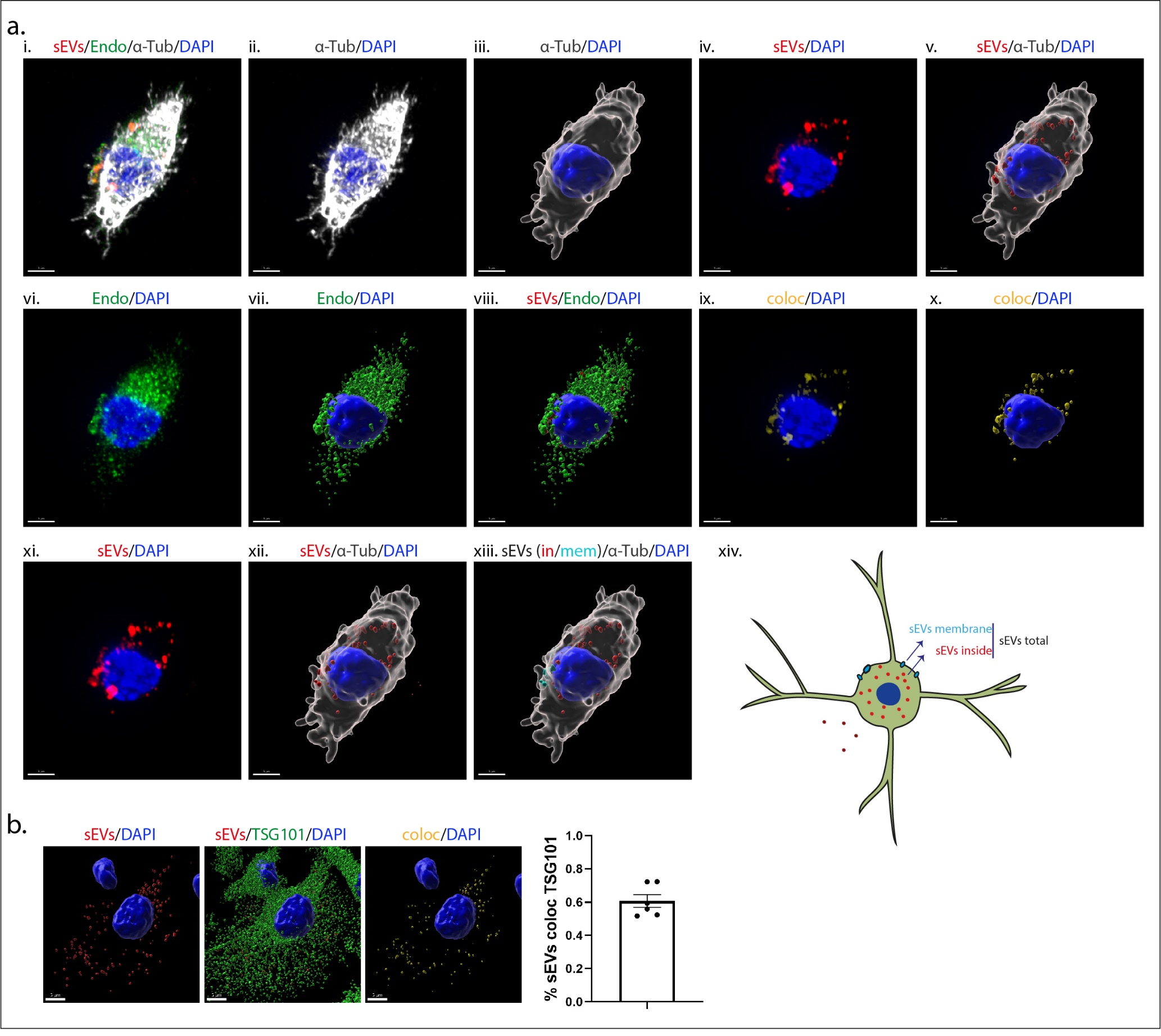
**

**Sup. Fig. 2. a. Analysis of fluorescent confocal images with the Imaris Imaging Software.** Confocal channels after deconvolution were imported into the Imaris Software (i). From the α-Tubulin (gray) and DAPI (blue) channels (ii), corresponding Imaris surfaces were created (iii). To measure total sEVs per cell, puncta of sEVs per cell and the mean volume of the individual puncta, α-Tubulin masked channels and surfaces corresponding to sEVs were created (iv and v). To measure colocalization of sEVs with Rab5 and Lamp1, first the same procedure was followed for Rab5 and Lamp1; corresponding α-Tubulin-masked channels were created, (vi), and converted into surfaces (vii). The fluorescent signal from the sEV, Rab5 and Lamp1 surfaces, (viii), was isolated into new masked channels that were imported into the “Coloc” module of Imaris in order to build the colocalization channel (ix) and respective surface (yellow) (x) for each pair of markers and evaluate the Manders’ coefficients of interest. In order to analyze the volume of internalized sEVs (the α-Τubulin-masked sEV surface already created consists of fully internalized sEVs and the fractions of membranous sEV puncta lying within the α-Tubulin surface; here we are only interested in the former), a surface based on the original sEV channel (red, xi), was created, (xii), and Imaris’ ‘’Distance transformation’’ function was used to compute the distance of its objects (sEV puncta) from the ‘’α-Tubulin’’ surface. Zero-valued puncta (‘’sEV total’’) in the resulting “Minimum distance” statistic correspond to sEVs that have entered the cell entirely (“sEV inside”) and those located on the plasma membrane and are in the process of entering (“sEV membrane”), (xiii, red and cyan blue, respectively, and in schematic illustration, xiv). The “sEV inside” puncta are characterized by being zero-valued in the “Maximum distance” statistic, also created from the “Distance transformation” function. Scale bar 3 μm. Schematic illustration of exo total, sEV in and sEV mem (xiv). **b.** Microglia cells were incubated with Dil-stained brain-derived sEVs (depicted in red) and internalization was monitored 6h post-incubation. Cells were fixed and immunostained with an antibody against TSG101 (green) while cell nuclei were stained with DAPI (blue). Confocal images were deconvolved and analyzed with the Imaris Imaging software. Colocalization of sEVs and TSG101 is depicted in yellow. The graph shows colocalization between EVs and TSG101 (Manders’ Colocalization Coefficient).


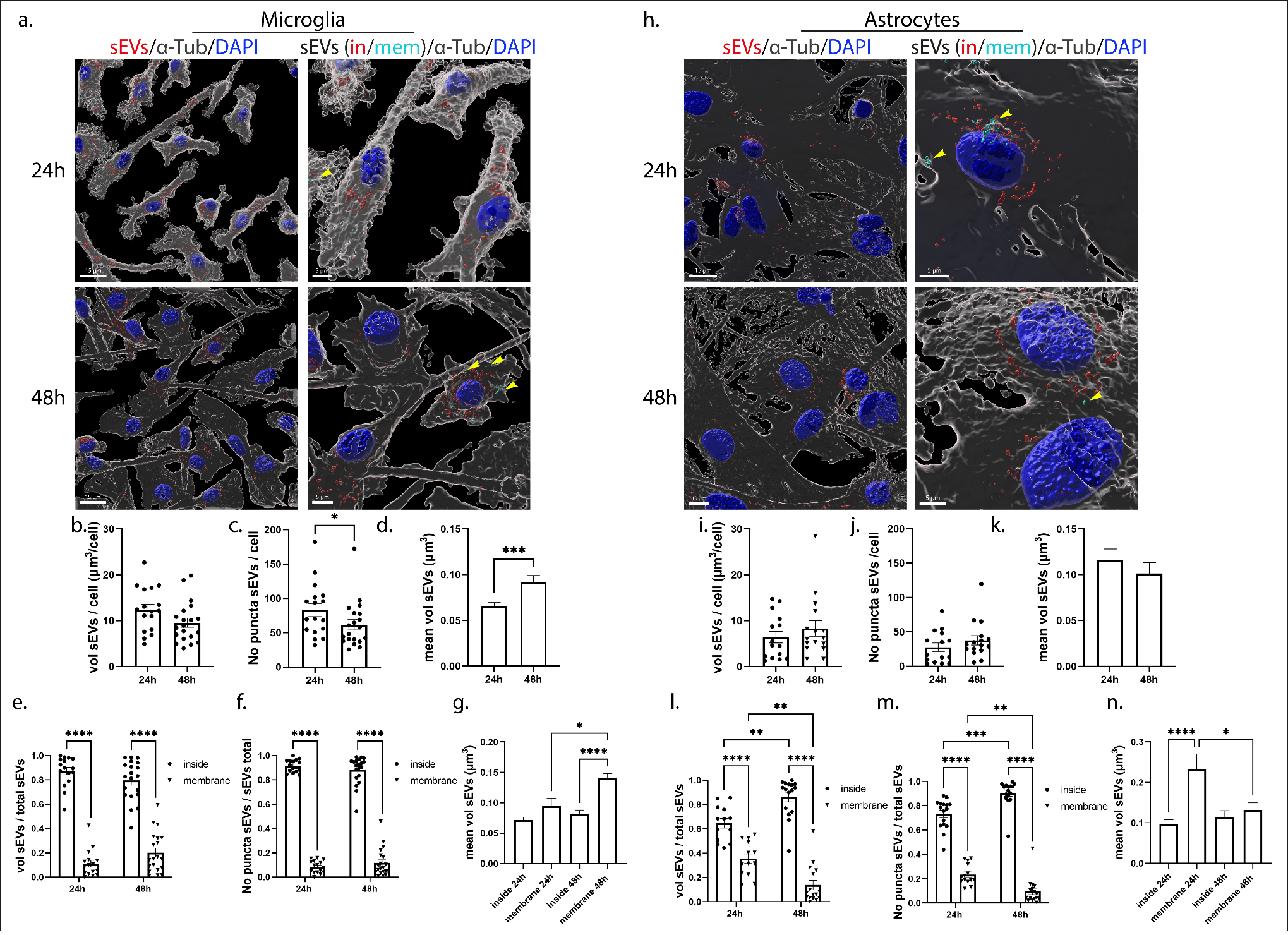


**Sup. Fig. 3.** **Endocytosis of brain-derived sEVs in primary microglia and astrocytes 24h and 48h post-treatment.** Primary cells were incubated with Dil-stained sEVs (depicted in red) for 24h, were washed, and internalization of sEVs was monitored at 24h and 48h of treatment. Cells were fixed and immunostained with an antibody against α-Tubulin (α-Tub) (gray) while cell nuclei were stained with DAPI (blue).­ Confocal images were deconvolved and analyzed with the Imaris Imaging software while sEVs were compartmentalized as cytoplasmic (sEV inside, red) or membranous (sEV membrane, cyan blue). Representative Imaris images depict the trafficking of sEVs masked with the tubulin surface (left panel, scale bar 15 μm) and in/mem sEVs (right panel, scale bar 5 μm) in microglia (a-g) and astrocytes (h-n) 24h and 48h post-addition. ‘’sEVs mem’’ were depicted with arrowheads. Graphs show the total volume of internalized sEVs per cell (b, i), the number of puncta per cell (c, j), the mean volume of sEVs (d, k), the ratio of the volume of sEVs (in/mem) per total volume (e, l), the ratio of the number of puncta (in/mem) per total number (f, m) and the mean volume of sEVs (in/mem) (g, n), in microglia and astrocytes respectively. Data are presented as the mean ± SEM of minimum 3 independent cell preparations, with at least two replicates per assay; Student's t-test was used for (b), (d) and (k), Mann-Whitney test for (c), (i) and (j), one-way ANOVA for (g) and (n), two-way ANOVA with Tukey’s correction for (e) and (l) and multiple t-test for (f) and (m). Statistical significance was set as *p < .05, **p < .01, ***p < .001, ****p < .0001.


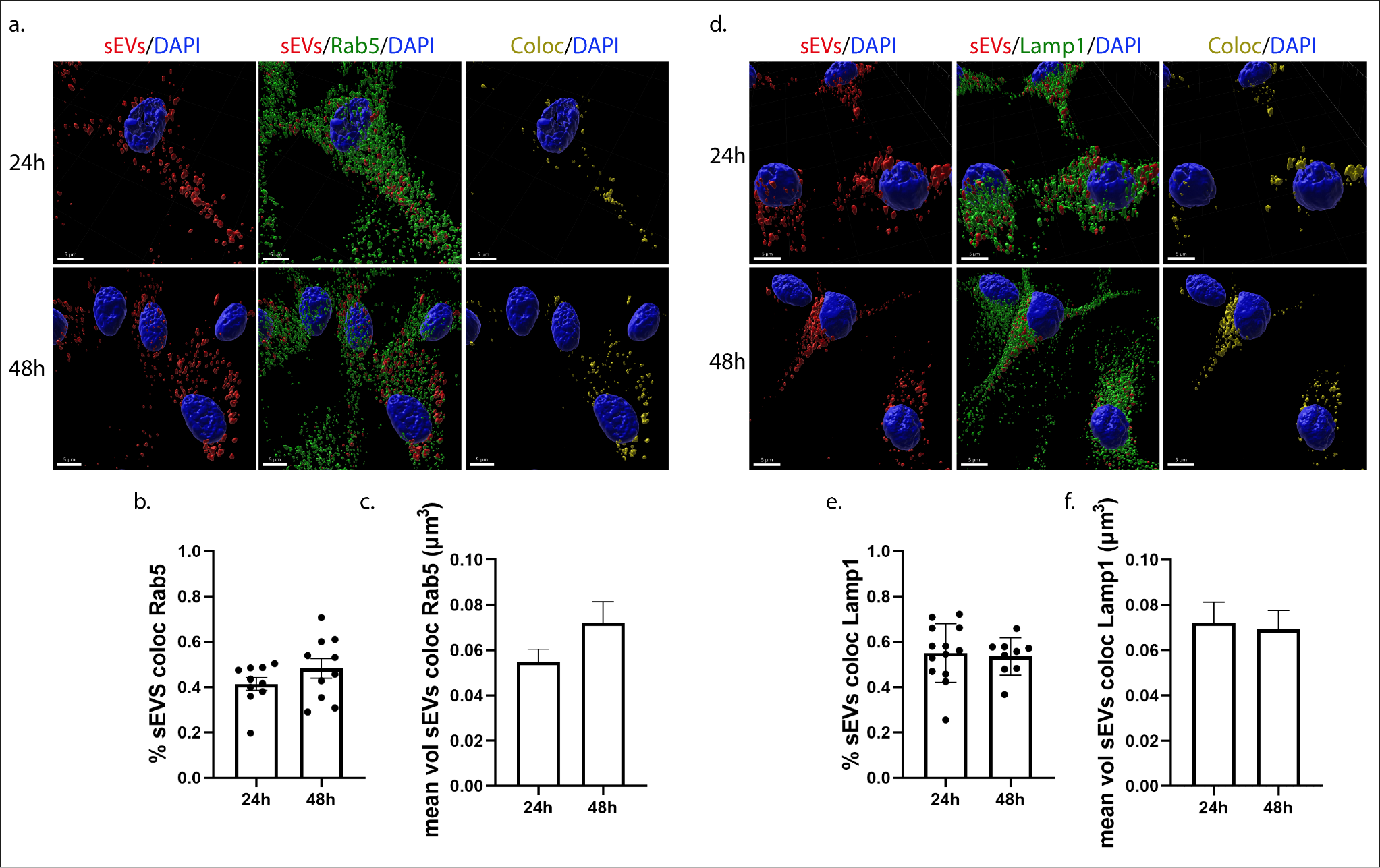


**Sup. Fig. 4. sEVs follow the endocytic pathway and are colocalized with Rab5 (EE) and Lamp1 (LE/Lysosomes) in primary microglia, at late time points.** Cells, incubated with Dil-labeled sEVs (red) for 24h, were washed and colocalization of sEVs with Rab5 and Lamp1 was monitored 24h and 48h post-treatment. Cells were fixed and immunolabeled for Rab5 or Lamp1 (green), α-Tubulin (α-Tub) (gray) and DAPI (blue). Representative Imaris images depict colocalization between sEVs and the endocytic markers, Rab5 (a-c) and Lamp1 (d-f), 24h and 48h post-addition. Scale bar 5 μm. Graphs show colocalization between sEVs and Rab5/Lamp1 (Manders’ Colocalization Coefficient) after 24h and 48h (b and e, respectively) of treatment as well as the mean volume of puncta colocalized with Rab5 (c) and Lamp1 (f) at different time points. Data are presented as the mean ± SEM of minimum 3 independent cell preparations, with more than 80 cells measured; Student's t-test was used, and statistical significance was set as *p < .05, **p < .01, ***p < .001, ****p < .0001.


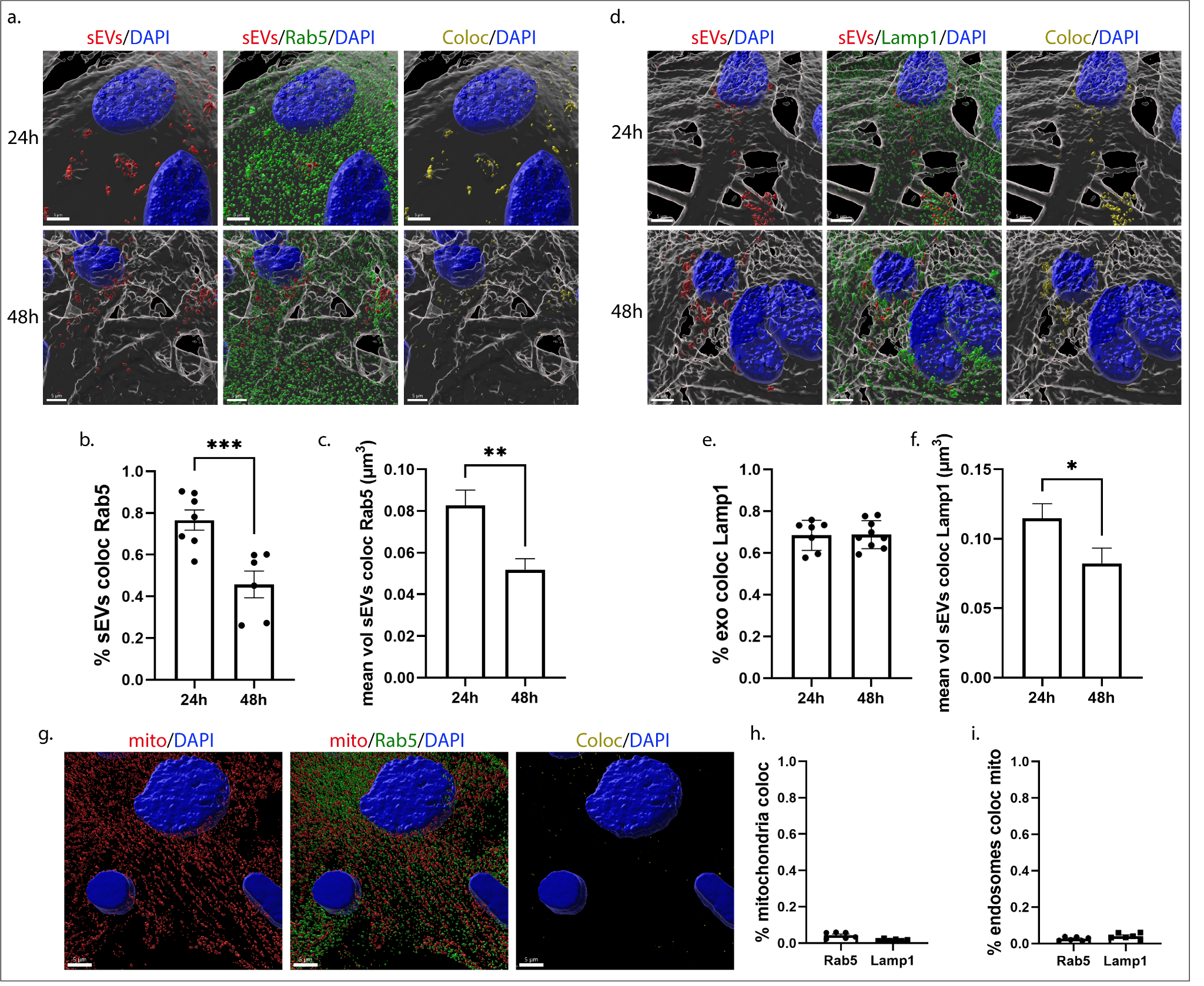


**Sup. Fig. 5. sEVs follow the endocytic pathway and are colocalized with Rab5 (EE) and Lamp1 (LE/Lysosomes) in primary astrocytes, at late time points.** Cells, incubated with Dil-labeled sEVs (red) for 24h, were washed and the endocytic trafficking of sEVs was monitored 24h and 48h post-treatment. Cells were fixed and immunolabeled for Rab5 or Lamp1 (green), α-Tubulin (α-Tub) (gray) and DAPI (blue). Representative Imaris images depict colocalization between sEVs and the endocytic markers, Rab5 (a-c) and Lamp1 (d-f), 24h and 48h post-addition. Scale bar 5 μm. Graphs show colocalization between sEVs and Rab5/Lamp1 (Manders’ Colocalization Coefficient) after 24h and 48h (b and e, respectively) of treatment as well as the mean volume of puncta colocalized with Rab5 (c) and Lamp1 (f) at different time points. Data are presented as the mean ± SEM of minimum 3 independent cell preparations, with more than 60 cells measured; Student's t-test was used, and statistical significance was set as *p < .05, **p < .01, ***p < .001, ****p < .0001. g. Astrocytes were labelled with a MitoTracker probe (depicted in red) and for Rab5. Representative Imaris images and graphs (h,i) show colocalization between mitochondria (mito) and Rab5. Scale bar 5 μm.


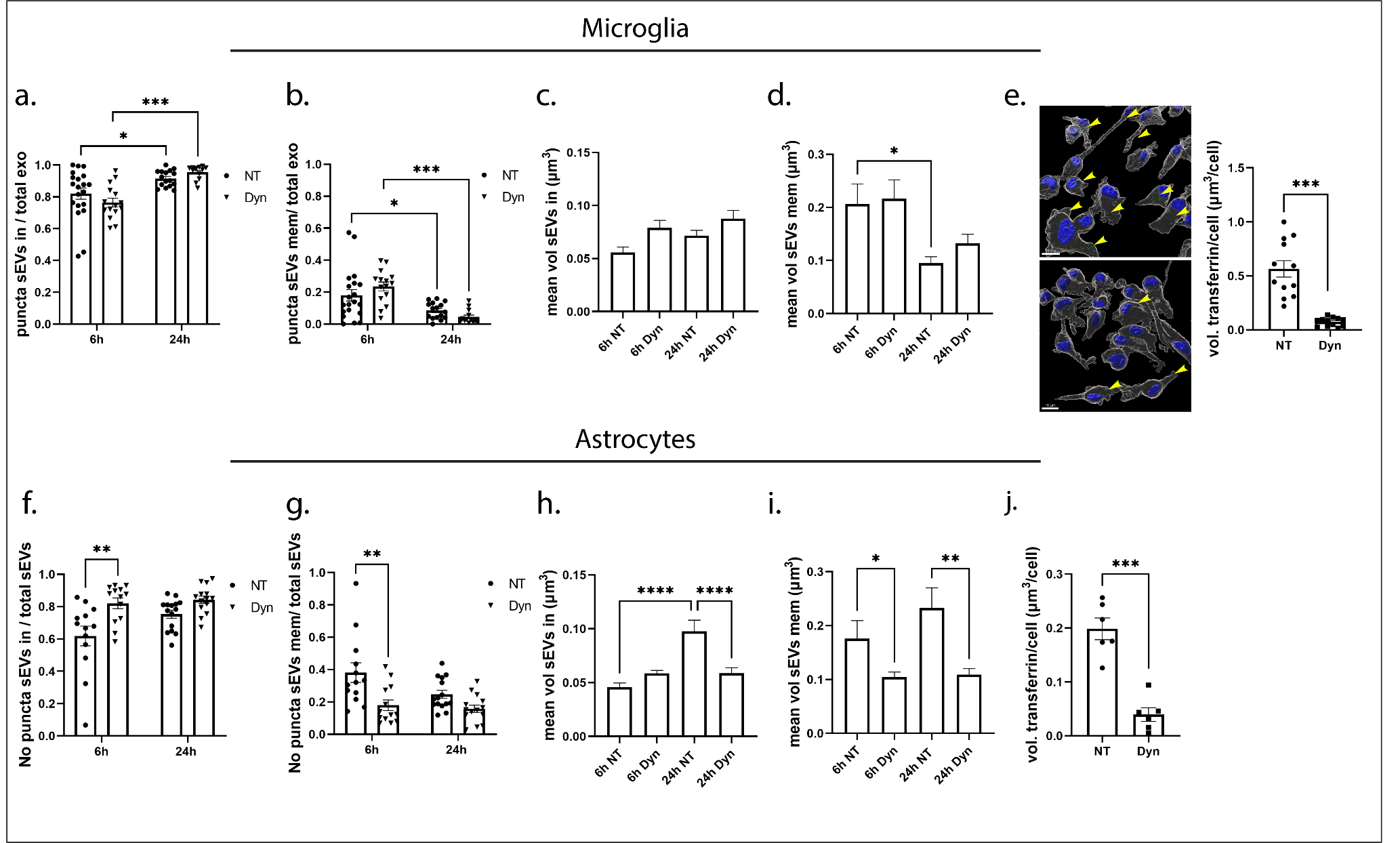
­

**Sup. Fig. 6. Uptake of sEVs in primary microglia and astrocytes following inhibition of the Dynamin-dependent endocytic pathway.** Graphs (related to Figure 5a and 6a) show the percentage of puncta, inside (a, f) and membrane (b, g) per total puncta, and the mean volume of sEVs, inside (c, h) or membrane (d, i), after 6h and 24h of treatment, with or without dynasore, in microglia and astrocytes, respectively. **e, j.** Microglia and astrocytes were incubated with 100 μM Alexa-Fluor 488 conjugated transferrin (depicted with arrowheads)­ for 5­­ and 30 min, respectively, and uptake was monitored in NT and Dyn treated cells. Graphs (e, j) show the volume of transferrin per cell. Data are presented as the mean ± SEM of minimum 3 independent cell preparations for a-d and f-I, and 2 independent cell preparations for e and j; Student’s t-test was used for (c) (j), one-way Anova for (d), (h) and (i) and multiple t-test for (a), (b), (f) and (g), and statistical significance was set as *p < .05, **p < .01, ***p < .001, ****p < .0001.


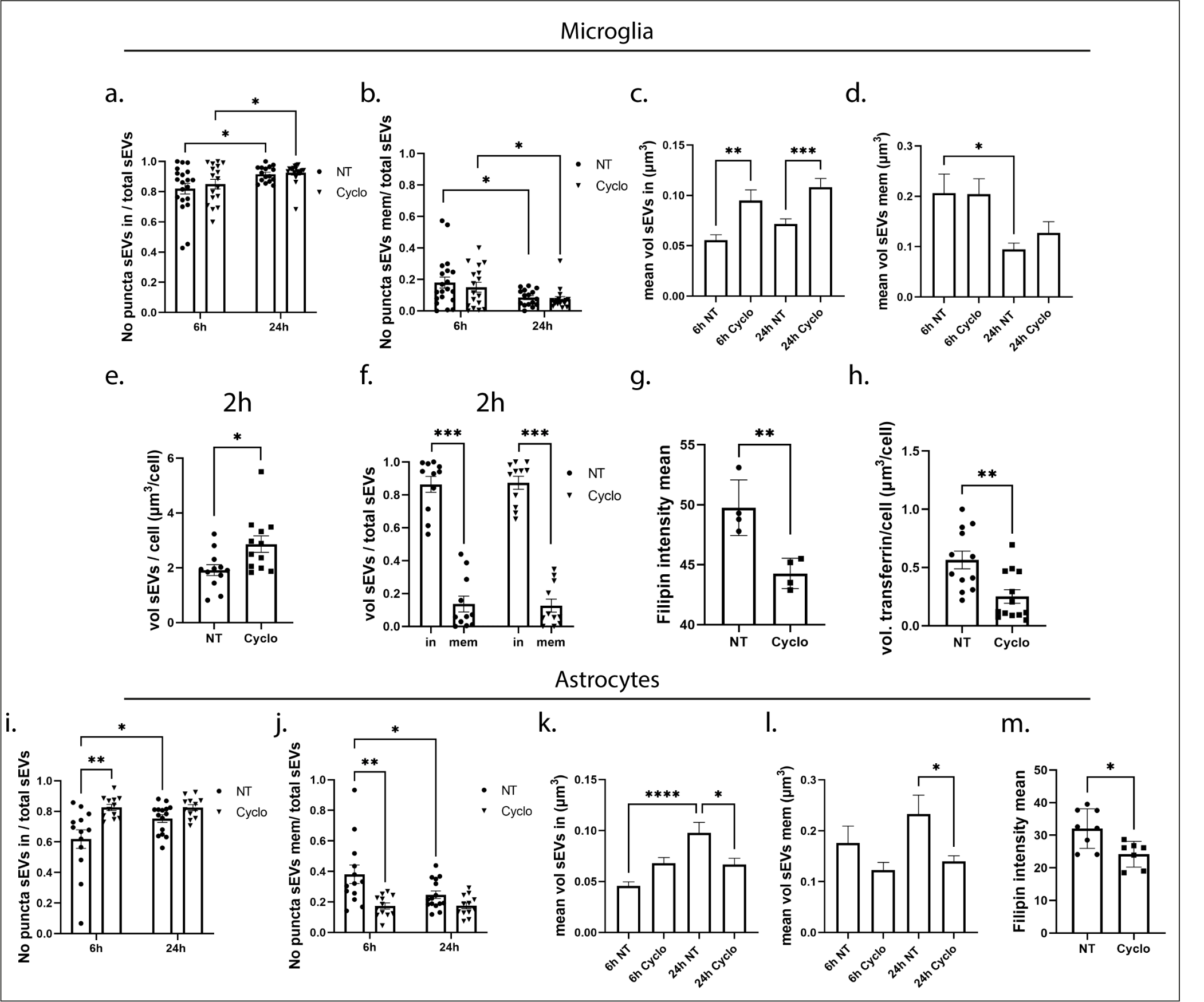


**Sup. Fig. 7. Internalization of sEVs in primary microglia and astrocytes upon inhibition of lipid raft-mediated endocytosis.** Graphs (related to Figure 7a and 8a) show the percentage of puncta, inside (a and i) and membrane (b and j), per total puncta, and the mean volume of sEVs, inside (c and k) or membrane (d and l), after 6h and 24h of treatment, with or without methyl-β-cyclodextrin (Cyclo), in microglia and astrocytes, respectively. **e, f.** Microglia were treated with Dil-labeled sEVs (red), in the absence (NT) or presence of methyl-β-cyclodextrin (Cyclo) for 2h. Graphs show the total volume of internalized sEVs per ce€(e), and the percentage of sEV volume, inside or membrane, per total volume, 2h post-incubation. **g, m.** Microglia and astrocytes were incubated with filipin, and graphs (g and m, respectively) show the mean intensity of filipin. **h.** Microglia was incubated with 100 μM Alexa-Fluor 488 conjugated transferrin for 5 min and uptake was monitored in NT and Cyclo treated cells. The graph (h) shows the volume of transferrin per cell. Data are presented as the mean ± SEM of minimum 3 independent cell preparations for a-d and i-l, and 2 independent cell preparations for e-h and m; Student’s t-test was used for (e), (g), (h), (m), one-way Anova for (c), (d), (f), (k) and (l) and multiple t-test for (a), (b), (i) and (j), and statistical significance was set as *p < .05, **p < .01, ***p < .001, ****p < .0001.


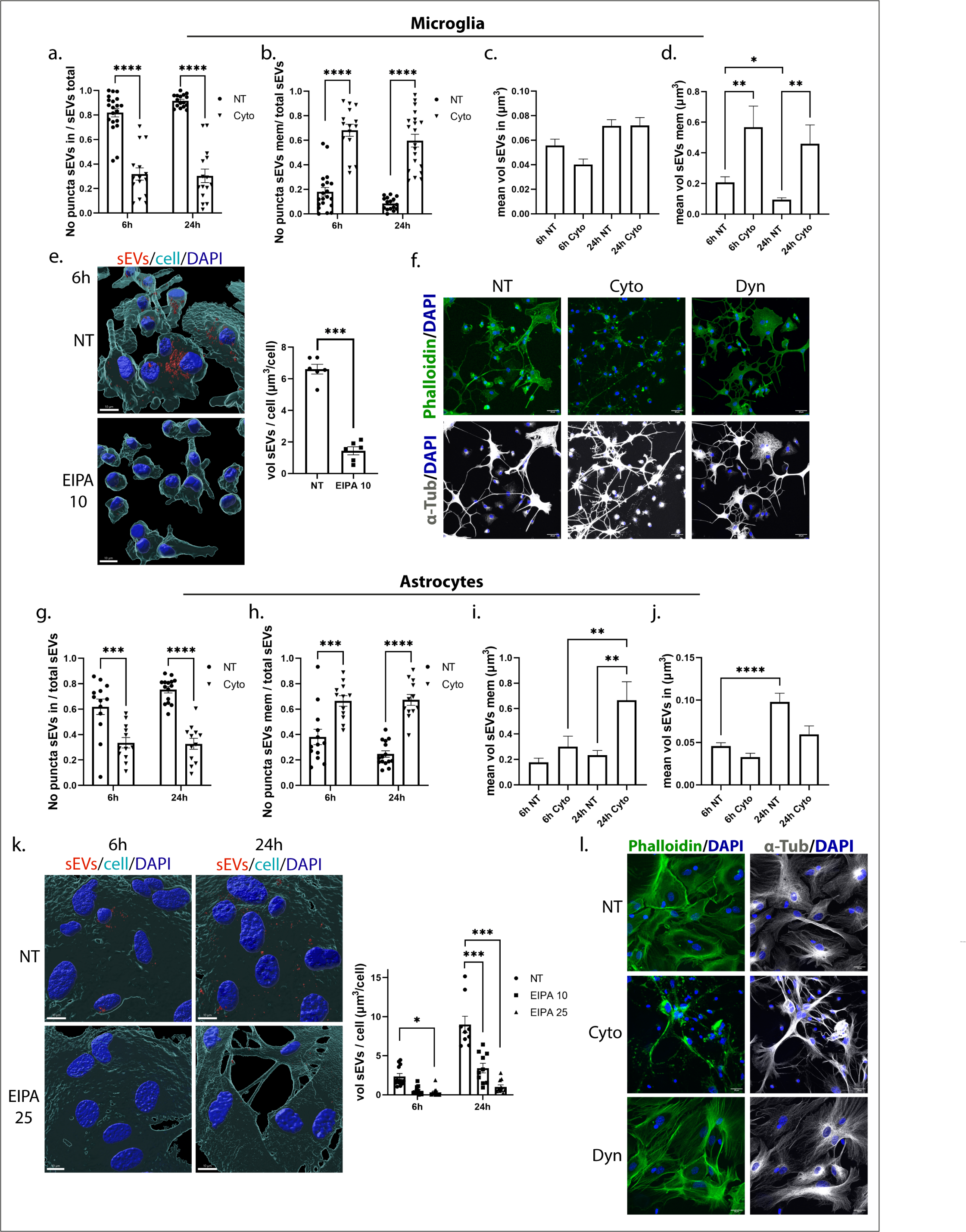


**Sup. Fig. 8. Internalization of sEVs in primary microglia and astrocytes through macropinocytosis.** Graphs (related to Figure 9a and 10a) show the percentage of puncta, inside (a and g) and membrane (b and h), per total puncta, and the mean volume of sEVs, inside (c and i) or membrane (d and j), after 6h and 24h of treatment, with or without Cytochalasin D (cyto), in microglia and astrocytes, respectively. **e, k.** Microglia and astrocytes, treated with Dil-labeled sEVs (red), in the absence (NT) or presence of different doses of ethyl-isopropyl amiloride (EIPA, 10 and 25 μM), were fixed and immunostained against α-Tubulin (α-Tub) (cell/cyan green) and DAPI (blue). Scale bar 10 μm. Graphs show the total volume of internalized sEVs per cell 6h post-incubation in microglia (e) and 6h and 24h post-incubation in astrocytes (k).­ **f, l.** Microglia (f) and astrocytes (l), in the absence or presence of Cyto or Dyn, were stained with Alexa Fluor 647 phalloidin (green), α-Tub (gray) and DAPI (blue). Scale bar 25 μm. Data are presented as the mean ± SEM of minimum 3 independent cell preparations for a-d and g-j, and 2 independent cell preparations for e, f, k and l; Student’s t-test was used for (e) and (k), one-way Anova was used for (c), (d), (i) and (j), two- way Anova for (a) and (b), and multiple t-test for (g) and (h), and statistical significance was set as *p < .05, **p < .01, ***p < .001, ****p < .0001.


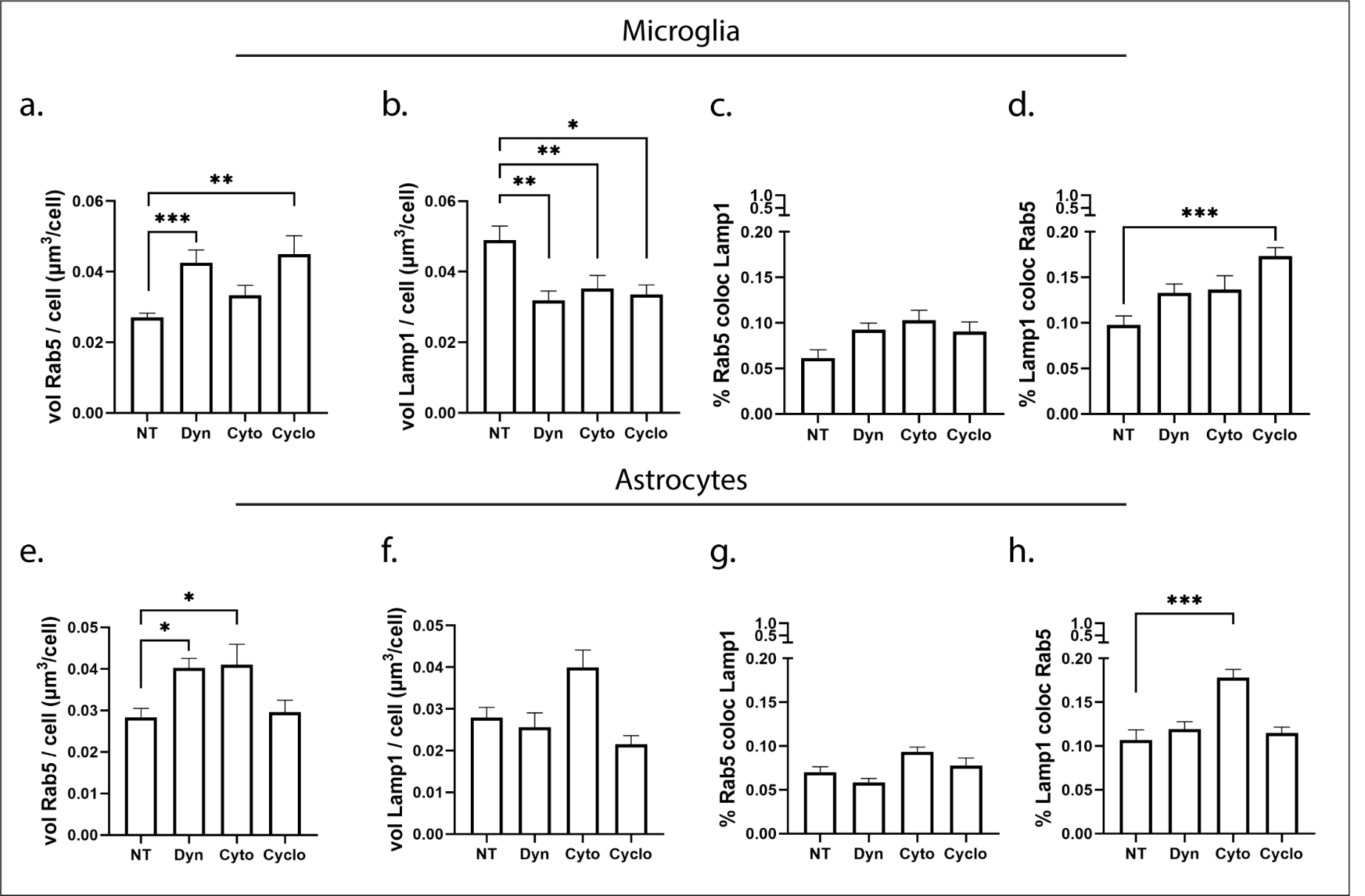


**Sup. Fig. 9. Endolysosomal maturation in primary microglia and astrocytes upon inhibition of the distinct endocytic pathways.** Microglia and astrocytes were fixed and stained against Rab5 and Lamp1. Graphs show the volume of Rab5 and Lamp1 per cell (a, b, e, f) in microglia and astrocytes, as well as the colocalization of the endosomal markers (c, d, g, h). Data are presented as the mean ± SEM of 2 independent cell preparations; Kruskal-Wallis test was used for (a) and (b), one-way Anova for (c), (d), (e), (f), (g) and (h), and statistical significance was set as *p < .05, **p < .01, ***p < .001, ****p < .0001.


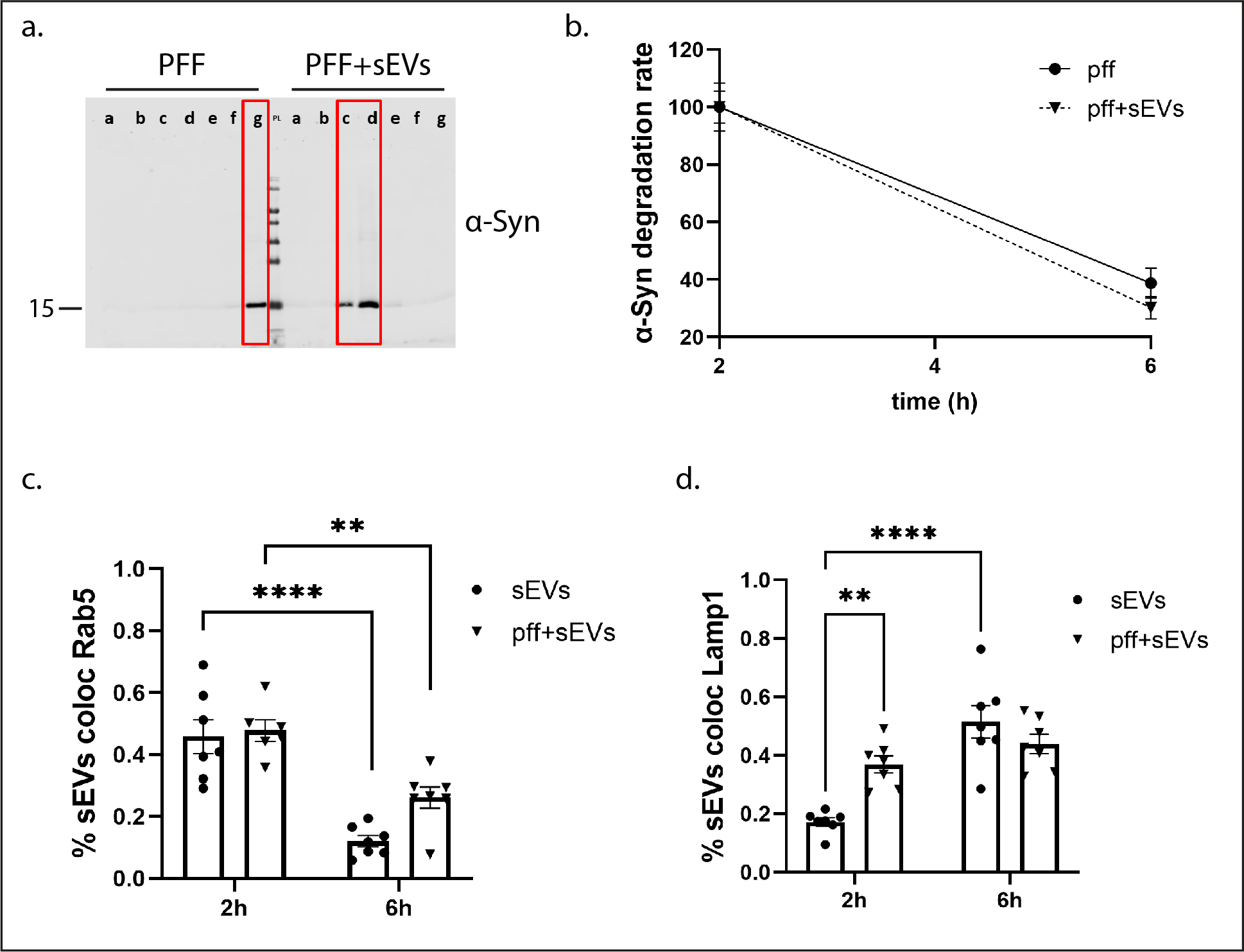


**Sup. Fig. 10. sEV-dependent α-Syn transmission in primary microglia**. **a.** PFF and PFF+sEVs were fractionated on a sucrose gradient column and fractions were subjected to immunoblotting against α-Syn. **b.** α-Syn degradation rate (related to Figure 11b) **c.** Graphs show colocalization of sEVs with Rab5 (c) and Lamp1 (d) at the different conditions (PFF, sEVs, PFF+sEVs) (related to Figure 12). Data are presented as the mean ± SEM of minimum 3 independent cell preparations, with at least two replicates per assay; two-way Anova was used, and statistical significance was set as *p < .05, **p < .01, ***p < .001, ****p < .0001.

**
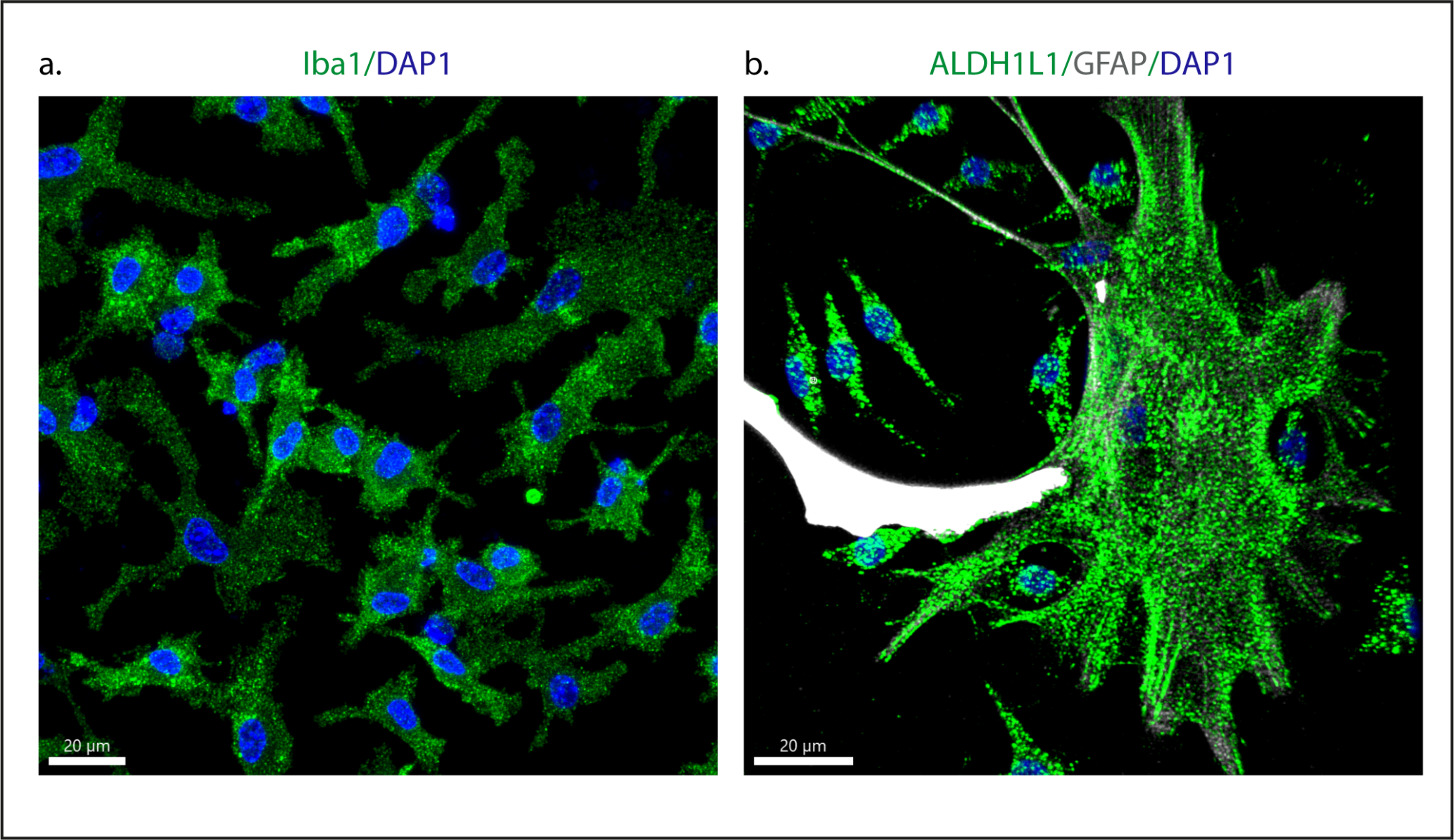
**

**Sup. Fig. 11**. Microglia cells were fixed and immunostained against Iba1 (a) and astrocytes against ALDH1L1 and GFAP. Cell nuclei were stained with DAPI (blue). Scale bar, 20 µm.
